# Early peripheral blood gene expression associated with good and poor 90-day ischemic stroke outcomes

**DOI:** 10.1186/s12974-022-02680-y

**Published:** 2023-01-23

**Authors:** Hajar Amini, Bodie Knepp, Fernando Rodriguez, Glen C. Jickling, Heather Hull, Paulina Carmona-Mora, Cheryl Bushnell, Bradley P. Ander, Frank R. Sharp, Boryana Stamova

**Affiliations:** 1 Department of Neurology, University of California at Davis, MIND Institute Biosciences Building Room 2417, 2805 50th Street, Sacramento, CA USA; 2 Division of Neurology, University of Alberta, Edmonton, AB Canada; 3 Wake Forest University School of Medicine, Winston Salem, NC USA

**Keywords:** Ischemic stroke, Outcomes, Gene expression, Transcriptome, WGCNA

## Abstract

**Background:**

This study identified early immune gene responses in peripheral blood associated with 90-day ischemic stroke (IS) outcomes.

**Methods:**

Peripheral blood samples from the CLEAR trial IS patients at ≤ 3 h, 5 h, and 24 h after stroke were compared to vascular risk factor matched controls. Whole-transcriptome analyses identified genes and networks associated with 90-day IS outcome assessed using the modified Rankin Scale (mRS) and the NIH Stroke Scale (NIHSS).

**Results:**

The expression of 467, 526, and 571 genes measured at ≤ 3, 5 and 24 h after IS, respectively, were associated with poor 90-day mRS outcome (mRS ≥ 3), while 49, 100 and 35 genes at ≤ 3, 5 and 24 h after IS were associated with good mRS 90-day outcome (mRS ≤ 2). Poor outcomes were associated with up-regulated genes or pathways such as IL-6, IL-7, IL-1, STAT3, *S100A12*, acute phase response, P38/MAPK, FGF, *TGFA*, *MMP9*, NF-kB, Toll-like receptor, iNOS, and PI3K/AKT. There were 94 probe sets shared for poor outcomes vs. controls at all three time-points that correlated with 90-day mRS; 13 probe sets were shared for good outcomes vs. controls at all three time-points; and 46 probe sets were shared for poor vs. good outcomes at all three time-points that correlated with 90-day mRS. Weighted Gene Co-Expression Network Analysis (WGCNA) revealed modules significantly associated with 90-day outcome for mRS and NIHSS. Poor outcome modules were enriched with up-regulated neutrophil genes and with down-regulated T cell, B cell and monocyte-specific genes; and good outcome modules were associated with erythroblasts and megakaryocytes. Finally, genes identified by genome-wide association studies (GWAS) to contain significant stroke risk loci or loci associated with stroke outcome including *ATP2B*, *GRK5*, *SH3PXD2A*, *CENPQ*, *HOXC4, HDAC9, BNC2*, *PTPN11*, *PIK3CG*, *CDK6,* and *PDE4DIP* were significantly differentially expressed as a function of stroke outcome in the current study.

**Conclusions:**

This study suggests the immune response after stroke may impact functional outcomes and that some of the early post-stroke gene expression markers associated with outcome could be useful for predicting outcomes and could be targets for improving outcomes.

**Supplementary Information:**

The online version contains supplementary material available at 10.1186/s12974-022-02680-y.

## Introduction

Changes in gene expression after ischemic stroke (IS) can potentially be used as biomarkers for causes of IS and predicting IS outcome [1–3]. Finding genes associated with long-term recovery after IS will improve our understanding of the pathways involved in recovery mechanisms, and may guide the search for treatment targets and early predictors of IS outcome [2, 4, 5].

Genetic risk factors have been associated with IS outcome, including *PTGIS*, *TBXAS1*, *IL6*, *BDNF*, *CYPC19*, *GPIIIa*, *P2RY1*, *ITGB3*, *PATJ*, *ADAM23*, *GRIA1*, *PARK2*, *ABCB5* and several cytochrome P450 genes [6–9]. Moreover, a number of clinical variables have been associated with long-term IS outcome including blood pressure, glucose levels/diabetes, atrial fibrillation, and hyperlipidemia in addition to age and sex [7]. Other variables shown to be associated with outcome include stroke severity, type of treatment, severe complications and stroke etiology [7, 10].

Predicting functional outcome in stroke is challenging partly because of the complexity of the condition and lack of highly accurate prognostic models. Clinical and demographic variables only explain a portion of the variance in long-term IS outcome. Thus, it is important to identify additional biomarkers to explain the remaining long-term outcome variance and to better understand the mechanisms of recovery following stroke. Thus, we have studied the peripheral blood transcriptome of patients after IS to discover genes and pathways that associate with 90-day outcomes as assessed using the modified Rankin Score (mRS) and NIH Stroke Scale (NIHSS). Some of these genes might eventually be useful for predicting functional outcome after IS and some may be targets for improving stroke outcomes.

## Materials and methods

### Study participants

Peripheral blood was drawn from IS patients at ≤ 3, 5, and 24 h (*n* = 36 participants, 108 samples) as part of the Combined Approach to Lysis Utilizing Eptifibatide and Recombinant Tissue-Type Plasminogen Activator (CLEAR) trial (NCT00250991 at www.Clinical-Trials.gov) [11]. IS participants were treated with recombinant tissue plasminogen activator (rt-PA) with or without eptifibatide after the within 3 h blood sample was obtained. After treatment, blood samples were drawn at 5 h and 24 h post-stroke onset. Though a total of 94 patients were studied in the CLEAR trial, we only had transcriptome data at all three time-points and all the needed variables—including 90-day outcomes, on 36 patients. The eligibility criteria for the 36 patients were the same as for the entire CLEAR Trial participants and included cortical strokes who were seen and enrolled within 3 h of their stroke onset. All of the exclusion and inclusion criteria are listed in the original publication [11].

Control participants included Vascular Risk Factor Control (VRFC) participants with at least one cardiovascular risk factor (hypertension, diabetes mellitus, hyperlipidemia) recruited from the Sex Age and Variation in Vascular functionalitY (SAVVY, Cheryl Bushnell PI) study (NCT00681681) (*n* = 18) [12]. Eligibility criteria for selecting controls were that they did not have any cerebrovascular disease and were matched for age, sex, and vascular risk factors. The institutional review board (IRB) at each site approved the study, and each patient or a proxy provided informed consent. Differences in demographic data between groups were analyzed using a two-tailed *t*-test and χ^2^ analysis where appropriate with *P* < 0.05 considered significant.

### Sample processing and data analysis

Whole blood was collected into PAXgene tubes (PreAnalytiX) and RNA processed as previously described [4]. Each RNA sample was processed and hybridized on Affymetrix Human U133 Plus 2.0 GeneChips (Affymetrix, Santa Clara, CA). Raw probe-level gene expression values imported into Partek Genomics Suite software (Partek Inc, St Louis, MO) were summarized to probe set-level using Median Polish summarization and normalized using robust multichip averaging (RMA) and our internal-gene normalization approach [4, 13].

The gene expression at ≤ 3 h, 5 h, and 24 h was associated with 90-day mRS outcome (modified Rankin Score, categorical variable), and the NIHSS (NIH Stroke Scale, continuous variable). The mRS participants with 90-day mRS scores of 0, 1, and 2 were dichotomized into a Good Outcome group (*n* = 26 participants, 78 samples), and participants with 90-day mRS of 3, 4, and 5 into a Poor Outcome group (*n* = 10 participants, 30 samples). This mRS variable is referred to as dichotomized mRS hereafter. No participant had the maximum mRS = 6 (deceased) at 90 days in this dataset.

#### Gene expression associated with 90-day dichotomized mRS

An ANCOVA identified genes whose expression was significantly associated with 90-day Good and Poor Outcomes (dichotomized mRS) at each time-point (≤ 3 h, 5 h, and 24 h) after IS compared to VRFC. The ANCOVA model for each time-point was *Y*_*i*_ = *μ* + Diagnosis (Poor Outcome, Good Outcome, VRFC) + Hypercholesterolemia + Hypertension + Diabetes + Age + Sex + *ε*_*i*_, where *Y*_*i*_ is gene expression at ≤ 3 h, 5 h or 24 h, *μ* is the common effect for the whole experiment, and *ε*_*i*_ is the random error. Age was a continuous variable, and Sex and vascular risk factors (Hypercholesterolemia, Hypertension and Diabetes) were considered as binary variables (Male, Female; Yes or No). A false discovery rate (FDR) corrected *P* < 0.05 and a fold change (FC) > ∣2∣ were considered significant for the Poor Outcome versus VRFC and Good Outcome versus VRFC. We used a less strict cut-off of (FC) > ∣1.3∣ and *P* < 0.05 when comparing IS patients with Poor versus Good 90-day mRS outcomes to increase numbers of genes per regulated pathway to identify the most significantly regulated pathways.

#### Gene expression associated with 90-day NIHSS

Separate analyses identified genes significantly correlated with 90-day NIHSS outcome using gene expression at ≤ 3 h, 5 h and 24 h. *P* < 0.005 was considered significant. The details of these methods are provided in the Additional file 6: Methods.

### Weighted gene co-expression network construction and analysis

Networks were generated using the Weighted Gene Co-Expression Network Analysis (WGCNA) package [14]. Separate weighted gene co-expression networks were generated for ≤ 3, 5, and 24 h gene expression following the methods in our recent studies [15]. The details of these analyses are provided in the Additional file 6: Methods (WGCNA-1).

### Identifying IS outcome-associated modules

Module–outcome associations for Good and Poor outcomes were determined using ANCOVA models in Partek Genomics Suite using the module’s eigengene values. The details of these methods are provided in the Additional file 6: Methods (WGCNA-2).

### Network visualization and hub gene identification

The v*isantPrepOverall* R function within WGCNA generated a list of intramodular gene connections with parameters numint = 10,000 and signed = TRUE [16, 17]. These connections were then imported into Cytoscape for network visualization [18, 19]. Nodes represent genes within the module and edge the connections between genes. Minimum weight cut-off for edges was adjusted for each network to generate a figure with a visually distinguishable number of nodes and connections.

### Cell-specific gene involvement

To identify enrichment in blood cell type-specific genes, differentially expressed gene lists and module gene lists were overlapped with lists of blood cell type-specific genes [20, 21]. The significance of list overlaps was assessed using hypergeometric probability testing (R function *phyper*; *P* < 0.05 considered significant).

### Pathway and gene ontology analyses

Ingenuity Pathway Analysis (IPA®, QIAGEN) was performed on all probe set lists as previously described [22] with *P* < 0.05 being considered significant. Details of the Pathway and Gene Ontology (GO) Analyses are provided in the Additional file 6: Methods (Pathway Analyses).

## Results

### Participant demographics

There were no statistically significant differences in age, sex, race, and vascular risk factors between IS participants and vascular risk factor controls (VRFC) (*P* < 0.05, Table 1), except between participants with poor outcome and VRFC for age. Therefore, we included age as a covariate in all the comparison analyses to account for the effect of age on gene expression. The median NIHSS was 10.5, 7.5, 6, 4, and 2 for ≤ 3 h, 5 h, 24 h, 5 days and 90 days post-IS. The median mRS at 90d was 2 (Q1 = 1, Q3 = 3, range: (0–5)). 26 participants had good 90-day mRS outcome (0–2), and 10 had Poor 90-day outcome (3–5). No participant had a 90-day mRS of 6 (deceased). Two of the IS patients included in our analyses developed symptomatic hemorrhagic transformation at 24 h by CT brain scan, with one having a good 90-day outcome and the other a poor 90-day outcome. Therefore, it is likely they did not substantially affect the findings for poor outcome vs. good outcome.

**Table 1:** Demographic and clinical characteristics of ischemic stroke (IS) patients and vascular risk factor controls (VRFC)

	IS patients (*n* = 36)	Good IS (*n* = 26)	Poor IS (*n* = 10)	VRFC (*n* = 18)	*P* value IS vs. VRFC	*P* value poor vs. good	*P* value good vs. VRFC	*P* value poor vs. VRFC
Age (years; mean ± SD)	64.3 ± 13	61.9 ± 13.3	70.5 ± 13.9	58.1 ± 5.6	0.06	0.07	0.27	0.0002
Women, *n* (%)	16 (44.5)	14	4	12 (66.7)	0.15	1	0.22	0.24
Race (%)					0.70	1	1	0.6
White	83.3	84.6	80	88.9				
Black	16.7	15.4	20	11.1				
Hypertension no. (%)	23 (63.9)	17 (65.4)	6 (60)	12(66.7)	1	1	1	1
Diabetes no. (%)	5 (13.9)	4 (15.4)	1(10)	6 (33.3)	0.15	1	0.27	0.36
Hypercholesterolemia no. (%)	7 (19.5)	6 (23.1)	1 (10)	8 (44.5)	0.1	0.65	0.19	0.09
NIHSS 3-h, median, (Q1, Q3)	10.5(6, 15.5)	10.5 (6, 15)	9.5 (7, 17)	–				
NIHSS 5-h, median, (Q1, Q3)	7.5 (5, 12)	6.5 (4, 10)	10 (7, 13)	–				
NIHSS 24-h, median, (Q1, Q3)	6 (3, 10)	4 (8, 2)	9 (6, 17)	–				
NIHSS 5-day, median, (Q1, Q3)	4 (1, 7.5)	2 (1, 4)	8.5 (6, 16)	–				
NIHSS 90-day, median, (Q1, Q3)	2 (0, 4)	1(0, 2)	7.5 (3, 9)	–				

*P* values represent the comparison between groups using a two-tailed *t*-test

*NIHSS* National Institutes of Health Stroke Scale, *Q* quartile

### Association of gene expression with 90-day poor mRS IS outcome

**Fig. 1:**
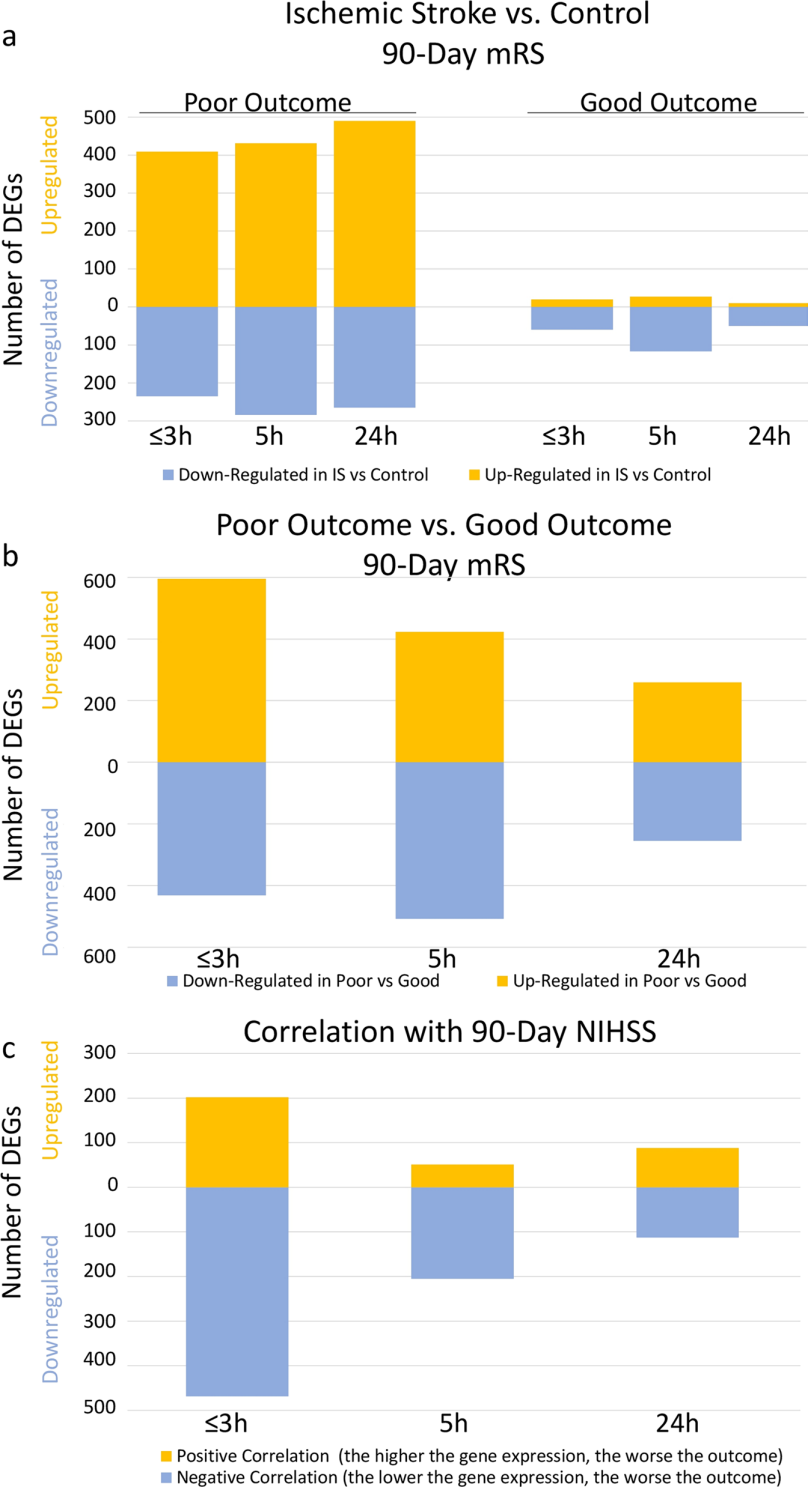
Numbers of up- and down-regulated differentially expressed genes (DEGs) at the three time-points ≤ 3 h, 5 h and 24 h after ischemic stroke. **a** Ischemic Stroke (IS) was compared to Vascular Risk Factor Controls (Control) for the Poor Outcome participants (mRS of 3–5 at 90 days) and for the Good Outcome participants (mRS of 0–2 at 90 days). **b** IS Poor Outcome participants were compared to the IS Good Outcome participants. **c** Numbers of DEGs that correlated with the 90-day NIHSS. The genes from the list for **a** passed FDR-corrected *P* value < 0.05 and a fold change (FC) >|2|. The list for the DEGs in **b** had a *P* value < 0.05 and a fold change (FC) >|1.3|. The list of the DEGs in **c** had *P* value < 0.005. Yellow represents numbers of up-regulated DEGs. Blue represents numbers of down-regulated DEGs. *IS* Ischemic Stroke, *mRS* modified Rankin Score, *NIHSS* National Institutes of Health Stroke Scale

**Fig. 2:**
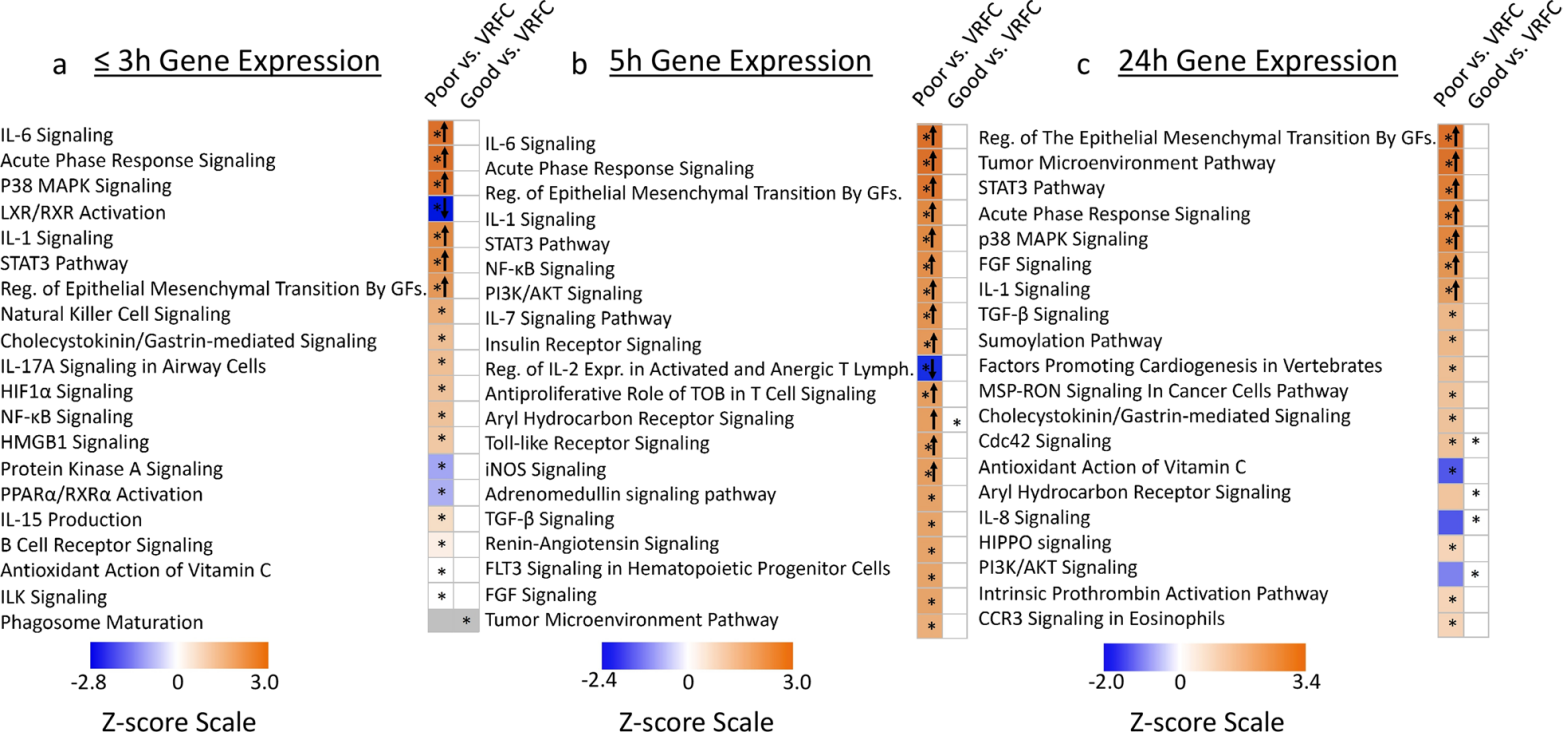
Top 20 most significant pathways enriched with differentially expressed genes (DEGs) in Poor IS Outcome (mRS 3–5) compared to Vascular Risk Factor Controls (Poor vs. VRFC) and in Good IS Outcome (mRS 0–2) compared to Vascular Risk Factor Controls (Good vs. VRFC). The top 20 most significant activation or suppression relevant pathways for these two comparisons are shown for the three time-points after stroke: **a** ≤ 3 h, **b** 5 h and **c** 24 h. Blue bars indicate pathway suppression (negative *Z*-score), and orange indicates activation (positive *Z*-score), with darker colors representing larger |*Z*-score|. ↑ (up arrow) represents *Z* ≥ 2 significant activation in the poor or good 90-day mRS IS outcome compared to VRFC. ↓ (down arrow) represents Z ≤ -2, significant suppression in the poor or good 90-day mRS IS outcome compared to VRFC. The asterisk * represents significantly enriched pathway (*P* < 0.05). White cells represent activity pattern prediction of Z = 0 (suppression or activation status cannot be predicated). Grey represents no activity pattern available for the pathway in the IPA knowledge base. *Reg*. regulation; *GFs*. growth factors; *Expr*. expression; *Lymph*. lymphocytes

**Fig. 3:**
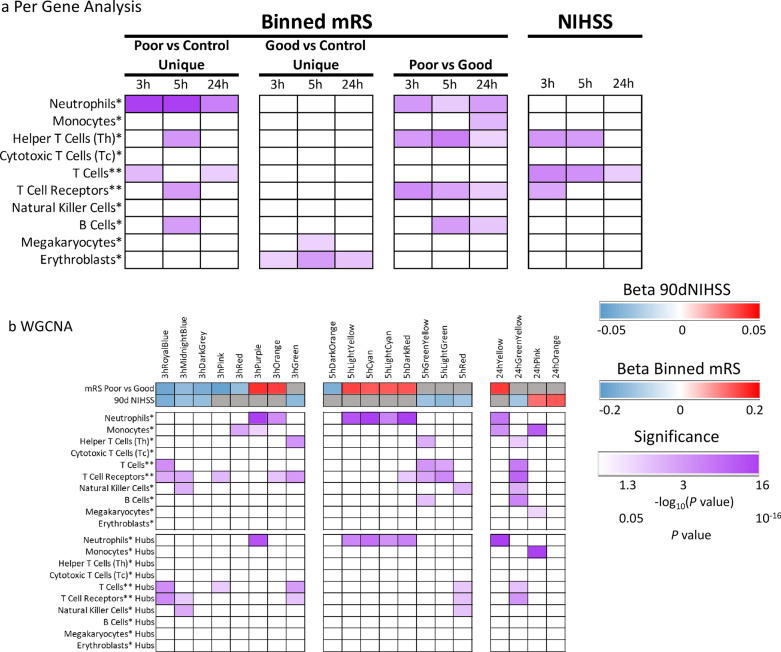
Enrichment in cell type-specific gene lists for the per-gene lists (**a)** and WGCNA modules (**b**). Purple shading represents − log_10_(*P* value) where 1.3 corresponds to a *P* value of 0.05. A higher − log_10_(*P* value) corresponds to lower (more significant—darker shades) *P* value. Non-significant hypergeometric probabilities are displayed as white cells. In **a**, the results are based on genes differentially expressed in poor 90d mRS IS outcome vs VRFC, good 90d mRS IS outcome vs VRFC, poor 90d mRS IS outcome vs good 90d mRS IS outcome, and genes correlating with 90d NIHSS. In **b** modules significant for 90-day outcome (mRS poor vs good, and NIHSS) are presented for the ≤ 3 h Network, 5 h Network, and 24 h Network. Blue indicates down-regulated and red up-regulated gene expression with worse outcomes via the beta coefficient for outcome in a linear regression on the module eigengene. Grey indicates modules not significantly associated with the outcome measure. Enrichment of hub gene lists in cell type-specific lists are presented at the bottom. The single asterisk * indicates cell type list from Watkins et al. [21] and the double asterisk ** indicates the cell type list was from Chtanova et al. [20]. Some of the identified Neutrophil genes might be expressed by other granulocytes, i.e., basophils and eosinophils

The data for the 3-h time-point are emphasized in the results and discussion because it is the only time at which the patients had not received any treatment; and genes that were regulated over all three times are also emphasized since they replicated. At ≤ 3 h post-IS, 644 probe sets (representing 467 genes) were differentially expressed in participants with poor 90-day outcome compared to VRFC (FDR-corrected *P* < 0.05, fold change (FC) >|2|) (Fig. 1a). Of these, 409 probe sets were up-regulated and 235 down-regulated (Fig. 1a, Additional file 5: Table S1A). The 644 probe sets were overrepresented in 47 pathways (Additional file 5: Table S2A). Top activated pathways included p38 MAPK, IL-6, IL-1 and STAT3. LXR/RXR was suppressed (Fig. 2a, represents only the top 20 most significantly enriched pathways with significant activation or suppression Z-scores, Additional file 5: Table S2A). Top overrepresented GO terms included B cell receptor signaling, phagocytosis, and immunoglobulin receptor binding, including Immunoglobin Heavy Constant genes such as *IGHG1*, *IGHG*3, *IGHA1*, *IGHA*2, *IGHD*, *IGHM*, and IGHV3-23 (FDR < 0.05) (Additional file 5: Table S3A). There was a significant enrichment with neutrophil-specific genes (63/467 genes (13.5%), *P*(overlap) < 1E−16) and T cell-specific genes (12/467 genes (2.6%), *P*(overlap) = 0.007) (Fig. 3a). Most neutrophil-specific genes (60/63) were up-regulated and T cell-specific genes down-regulated in participants with poor 90-day outcomes.

At 5 h post-IS, 715 probe sets (526 genes) were differentially expressed in participants with poor outcome compared to VRFC (FDR-corrected *P* < 0.05, FC >|2| (Fig. 1a). Of these, 431 probe sets were up-regulated and 284 down-regulated (Fig. 1a, Additional file 5: Table S1B). The 715 probe sets were overrepresented in 106 pathways (Additional file 5: Table S2B). Activated pathways included iNOS, Toll-like Receptor, IL-1, -6 and -7, NF-kB and STAT3 signaling; and regulation of IL-2 Expression was suppressed (Fig. 2b, represents only the top 20 most significantly enriched pathways with significant activation or suppression Z-scores, Additional file 5: Table S2B). There was enrichment in neutrophil-specific genes (76/526 genes (14.5%), *P*(overlap) < 1E−16); in T helper cell and T cell receptor signaling-specific genes (5/526 genes (1.0%), *P*(overlap) = 1E−04 and 14/526 genes (2.7%), *P*(overlap) = 5E−04, respectively); and in B cell-specific genes (15/526 genes (2.9%), *P*(overlap) = 6E−04) (Fig. 3a). Most neutrophil-specific genes were up-regulated (74/76), while T cell- (14/19) and B cell-specific genes (14/15) were down-regulated in poor outcome participants.

At 24 h post-IS 755 probe sets (571 genes) were differentially expressed between poor outcome and VRFC participants. The data for these analyses are provided in Additional file 6: Results and in Figs. 1a, 2c, 3a and Additional file 5: Tables S1C, S2C, S3B.

**Fig. 4:**
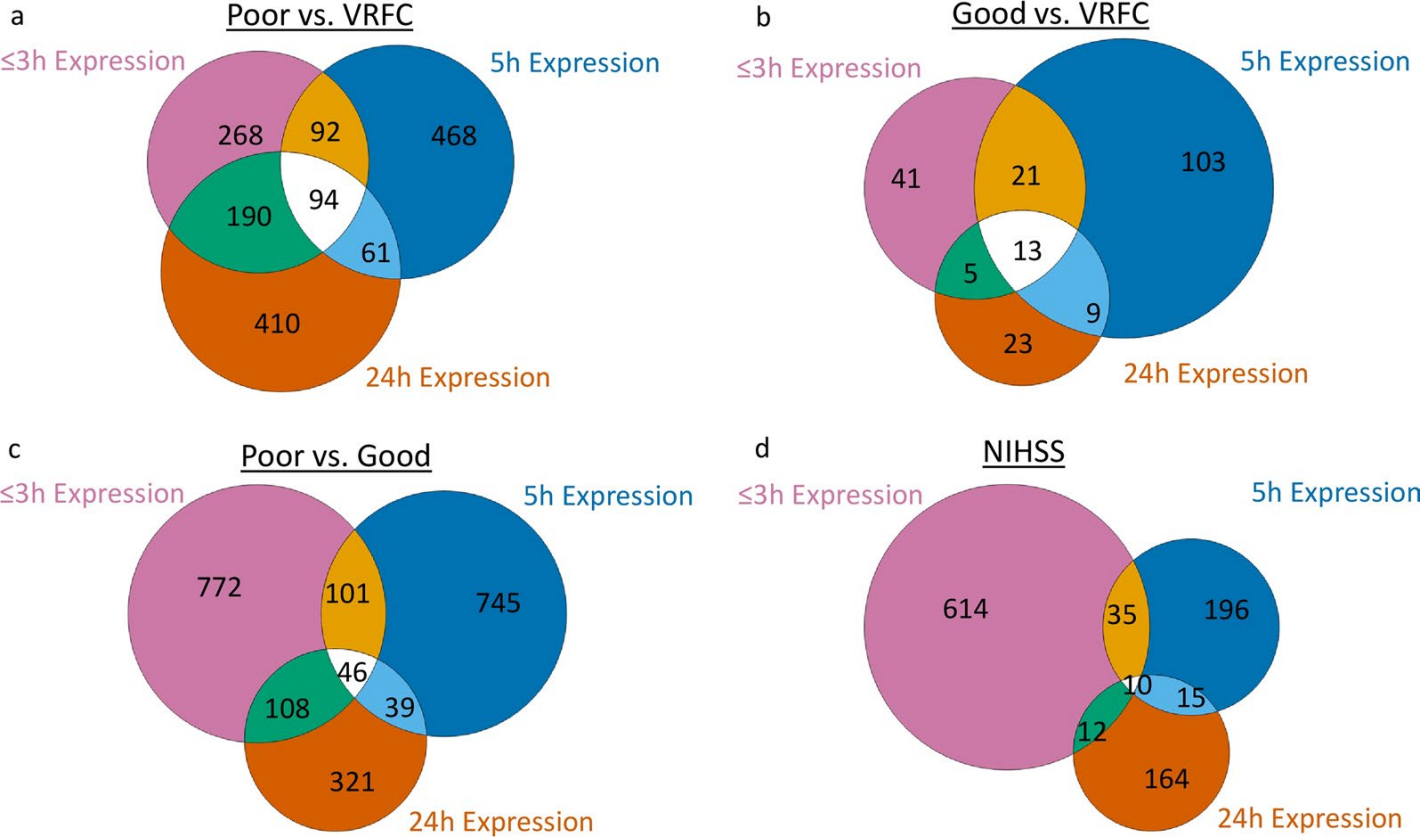
Venn diagram of the number of differentially expressed (DE) probe sets at each of the three time-points (≤ 3 h, 5 h and 24 h) post-stroke. **a** Numbers of DE probe sets between ischemic stroke (IS) participants with poor 90-day mRS outcomes compared vascular risk factor controls (VRFC). **b** Numbers of DE probe sets between IS participants with good 90-day mRS outcome compared to VRFC. **c** Numbers of DE probe sets between participants with poor 90-day mRS outcome compared to participants with good 90-day mRS outcome. **d** Numbers of DE probe sets that correlated with the 90-day National Institutes of Health Stroke Scale (NIHSS)

There were 94 probe sets (representing 78 genes) that were consistently differentially expressed at the three time-points post-IS (≤ 3 h, 5 h and 24 h) between poor 90-day mRS outcome and VRFC (FDR-corrected *P* < 0.05, FC >|2|) (Fig. 4a). Of these, 60 probe sets were up-regulated and 34 down-regulated at all three time-points (Additional file 5: Table S1D). The 94 probe sets were overrepresented in 31 pathways. Among the top pathways, IL-17 Signaling, Acute Phase Response Signaling and Natural Killer Cell Signaling were activated (*Z* ≥ 2) (Additional file 5: Table S2D). Genes that were consistently differentially expressed between IS with poor 90-day mRS outcome and controls over the three time-points were enriched in neutrophil-specific genes (15/78 genes (19.23%), *P*(overlap) = 1.41E−07) and were also significantly overlapping with genes that we have been shown in our previous study [15] to correlate with intracerebral hemorrhage volume (16/78 genes (20.51%), *P*(overlap) = 8.17E−12) and absolute peri-hematomal edema volume (8/78 genes (10.26%), *P*(overlap) = 7.88E−06) (data not shown).

### Association of gene expression with good 90-day mRS outcome

At ≤ 3 h post-IS, 80 probe sets (representing 49 genes) were differentially expressed in participants with good outcome compared to VRFC (FDR-corrected *P* < 0.05, FC >|2|) (Fig. 1a, Additional file 5: Table S1A). Of these, 20 probe sets were up-regulated and 60 down-regulated (Fig. 1a, Additional file 5: Table S1A). The 80 probe sets were overrepresented in 12 pathways including Phagosome Maturation, PTEN Signaling and Epithelial Adherens Junction (Additional file 5: Table S2A). There was significant enrichment in Erythroblast-specific genes (3/49 genes (6.1%), *P*(overlap) = 0.03) (Fig. 3a).

At 5 h post-IS, 146 probe sets (100 genes) were differentially expressed between good outcome and VRFC participants (FDR-corrected *P* < 0.05, FC >|2| (Fig. 1a). Of these, 29 probe sets were up-regulated and 117 down-regulated (Fig. 1a, Additional file 5: Table S1B). The 146 probe sets were overrepresented in 9 pathways (Additional file 5: Table S2B), such as NRF2- Oxidative Stress Response and RhoGDI Signaling with significant enrichment in Erythroblast-specific genes (7/100 genes (7.0%), *P*(overlap) = 7E−04) and Megakaryocyte-specific genes (4/100 (4.0%), *P*(overlap) = 4E−02) (Fig. 3a).

At 24 h post-IS, 50 probe sets (35 genes) were differentially expressed between good outcome and VRFC participants. The details of these analyses are provided in Additional file 6: Results and in Figs. 1a, 2c, 3a and Additional file 5: Tables S1C, S2C.

There were 13 probe sets (representing 10 genes) that were consistently differentially expressed at all three time-points post-IS between good 90-day mRS outcome and VRFC (FDR-corrected *P* < 0.05, FC >|2|) (Fig. 4b). All 13 probe sets were down-regulated at all three time-points (Additional file 5: Table S1E). They were overrepresented in 17 pathways including IL-8 Signaling, CD27 Signaling in Lymphocytes, VEGF Signaling and Apoptosis (Additional file 5: Table S2E).

### Direct comparison of 90-day poor vs. good mRS outcome

At ≤ 3 h post-IS, 1027 probe sets (709 genes) were differentially expressed between participants with poor and good 90-day mRS outcome with *P* < 0.05 and FC >|1.3|. 432 probe sets were down-regulated, 595 were up-regulated in poor vs. good outcome patients (Fig. 1b, Additional file 5: Table S1A). The 1027 probe sets were overrepresented in 62 pathways, with three activated (IL-1 and IL-6 signaling (Additional file 1: Fig. S1) and Remodeling of Epithelial Adherens Junctions (Additional file 5: Table S2A)) and three suppressed (ICOS-ICOSL Signaling in T Helper Cells, Calcium-induced T Lymphocyte Apoptosis, and T Cell Receptor Signaling) (Additional file 1: Fig. S1 and Additional file 5: Table S2A). There was also a significant enrichment in neutrophil-specific genes (46/709 genes (6.5%), *P*(overlap) = 2E−04); and in T helper cell-specific and T cell receptor and signaling-specific genes (5/709 genes (0.7%), *P*(overlap) = 4E−04 and 21/709 genes (3.0%), *P*(overlap) = 4E−06, respectively) (Fig. 3a). Notably, 45/46 neutrophil-specific genes were up-regulated, while 20/26 T cell-specific genes were down-regulated in participants with poor compared to participants with good 90-day functional outcome.

At 5 h post-IS, 931 probe sets (658 genes) were differentially expressed between poor outcome and good 90-day mRS outcome with *P* < 0.05 and FC >|1.3| (Fig. 1b). Of these, 508 were down-regulated, and 423 up-regulated in poor vs. good outcome (Fig. 1b, Additional file 5: Table S1B). They were overrepresented in 56 pathways (Additional file 5: Table S2B), with two activated including B Cell Receptor Signaling and five suppressed (ICOS-ICOSL Signaling in T Helper Cells, Th2 Pathway, and T Cell Receptor Signaling, and Role of NFAT in Regulation of the Immune Response). Several T cell-related pathways were overrepresented in the gene list (Additional file 5: Table S2B, Additional file 1: Fig. S1). In addition, there was significant enrichment in neutrophil-specific genes (35/658 genes (5.3%), *P*(overlap) = 0.02); B cell-specific genes (17/658 genes (2.6%), *P*(overlap) = 8E−04); and T helper-specific and T cell receptor and signaling-specific genes (8/658 genes (1.2%), *P*(overlap) = 6E−08 and 15/658 genes (2.3%), *P*(overlap) = 2E−03, respectively) (Fig. 3a). The neutrophil-specific genes were up-regulated in poor outcome (except *CCR3*), while T cell-specific genes were down-regulated in poor outcome, except five genes-*SOS2*, *CBL*, *SNTB2*, *APBB1IP* and *PRKCB*, which were up-regulated in poor outcome.

At 24 h post-IS, 514 probe sets (363 genes) were differentially expressed between poor vs. good 90-day outcome IS patients with *P* < 0.05 and FC >|1.3|. Of these 255 were negatively regulated, and 259 were positively regulated (Fig. 1b, Additional file 5: Table S1C). They were overrepresented in 61 pathways with one being suppressed (ICOS-ICOSL Signaling in T Helper Cells) (Additional file 1: Fig. S1-displays only the top 20 most significant activation or suppression relevant pathways, Additional file 5: Table S2C). Several immune response-related pathways were overrepresented among the 363-gene list, such as Th1 and Th2 Activation Pathway, T Helper Cell Differentiation, Interferon Signaling, and Role of JAK family kinases in IL-6-type Cytokine Signaling (Additional file 5: Table S2C). In addition, there was a significant enrichment in neutrophil-specific genes (26/363 genes (7.2%), *P*(overlap) = 1E−03); in B cell-specific genes (9/363 genes (2.5%), *P*(overlap) = 1.7E−02); in T helper-specific, and T cell receptor and signaling-specific genes (2/363 genes (0.6%), *P*(overlap) = 4.4E−02 and 8/363 genes (2.2%), *P*(overlap) = 2.2E−02, respectively); and in monocyte-specific genes (9/363 genes (2.5%) genes, *P*(overlap) = 6E−03) (Fig. 3a). Most of the neutrophil cell-specific genes were up-regulated (21/26), while most of the T cell-specific genes (9/10) and B cell-specific genes (8/9) were down-regulated in participants with poor vs. participants with good 90-day functional outcome.

There were 46 probe sets (representing 32 genes) that were consistently differentially expressed between poor 90-day IS outcome and good 90-day outcome over the three time-points (*P* < 0.05, FC >|1.3|) (Fig. 4c). Of these, 15 probe sets were up-regulated and 31 probe sets were down-regulated at all time-points. In addition, the *ZNF551* gene was up-regulated at ≤ 3 h while down-regulated at 5 h and 24 h (Additional file 5: Table S1F). The 46 probe sets were overrepresented in 30 pathways such as immune-related pathways, Calcium-induced T Lymphocyte Apoptosis, T Helper Cell Differentiation, NUR77 Signaling in T Lymphocytes and ICOS-ICOSL Signaling in T Helper Cells (Additional file 5: Table S2F).

### Association of gene expression with 90-day NIHSS

There were 671, 256 and 201 probe sets at ≤ 3, 5 and 24 h after IS, respectively, that associated with 90-day NIHSS. Of these were 10 probe sets that were associated with 90d NIHSS at all three time-points. The data from these analyses are described in Additional file 6: Results and in Figs. 1c, 3a, 4d, Additional file 2: Fig. S2 and Additional file 5: Tables S1A−S2A, S1B−S2B, S1C−S2C, S1G−S2G.

### Gene expression modules associated with poor vs. good 90-day outcomes following IS

**Fig. 5:**
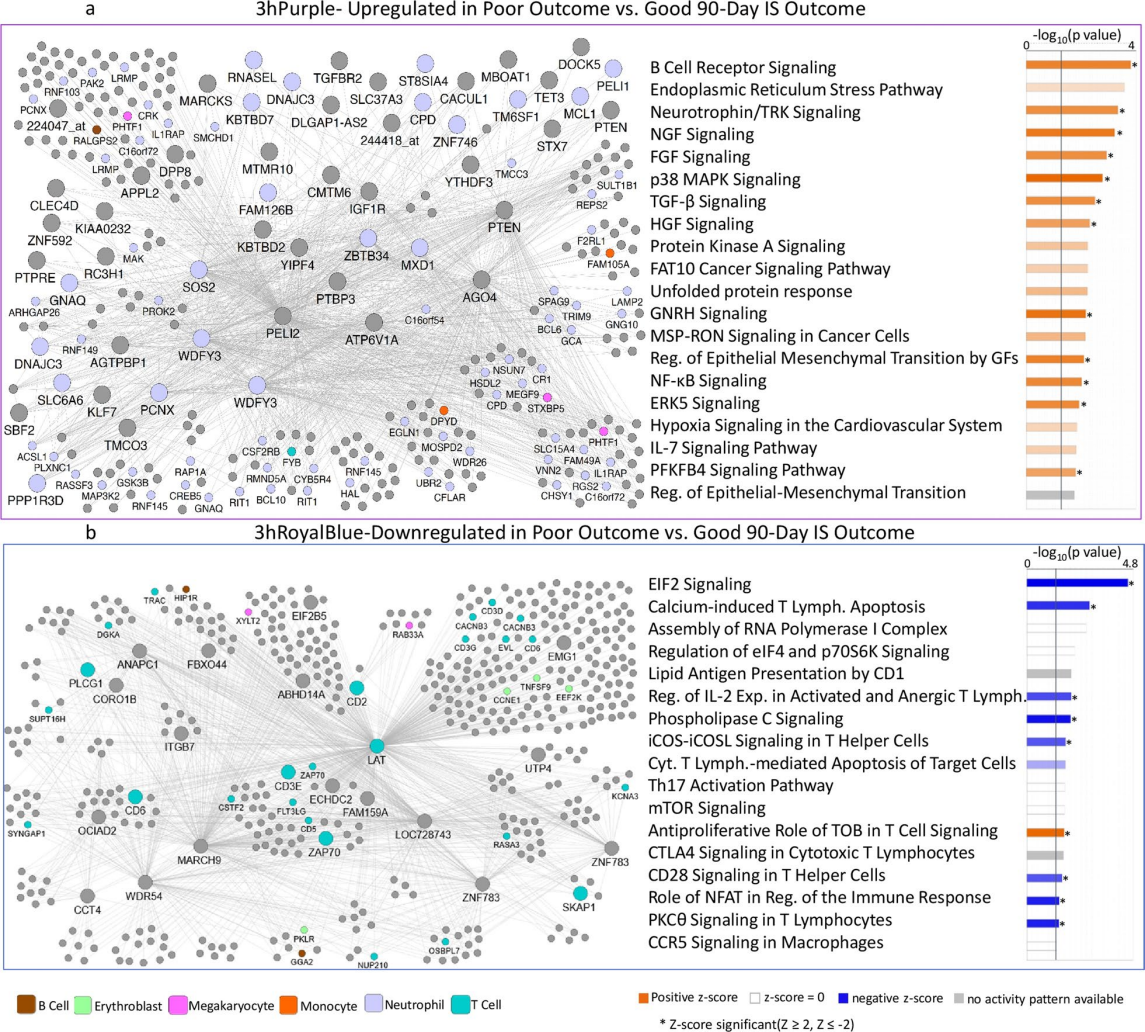
Network diagram (**a** left panel) and Pathway Enrichment (**a** right panel) for the outcome-significant (mRS poor vs. good) for the 3hPurple module. In the **a** left panel, the network diagram shows the connectivity of hubs and genes within the module. Nodes represent genes within the module and edges represent connections based on co-expression between genes. Larger nodes with large labels are hub genes, representing potential master regulators. Genes are grey by default and colored if they are cell type specific. In the **a** right panel, the top 20 most significant relevant pathways are displayed. The significance threshold (*P* = 0.05) corresponds to the vertical black line. Blue shading indicates suppression and orange activation with darker colors representing larger |*Z*-score|. Grey represents no activity pattern available for the pathway in the IPA knowledge base. An asterisk * represents statistically significant activation or suppression (*Z* ≥ 2 or *Z* ≤ − 2) in poor outcome compared to good outcome. In **b** the Network diagram (**b** left panel) and Pathway Enrichment (**b** right panel) for the outcome-significant (mRS poor vs. good) for the 3hRoyalBlue module. The *LAT* gene is colored as T cell-specific though it is expressed in megakaryocytes and T cells. White bars represent activity pattern prediction of *Z* = 0 (suppression or activation status cannot be predicated). Other details of this figure are identical to those in **a**. *Reg.* regulation, *GFs.* growth factors, *Expr.* expression, *Lymph.* lymphocytes

WGCNA was run on 28,686 Affymetrix probe sets for 36 IS participants, with separate WGCNA runs generated for each time-point (≤ 3 h, 5 h, and 24 h). Modules significantly associated with IS outcome such as 3hPurple, 3hRoyalBlue, 5hCyan, 24hYellow and 24hGreenYellow, and the canonical pathways significantly enriched in each module are presented in Figs. 3b, 5a, 5b, Additional file 3: Fig. S3, Additional file 4: Fig. S4. Table 2 lists the hub genes for the 3 h module and Additional file 5: Table S4 lists the hub genes for the 5 h and 24-h time-points.

**Table 2:**
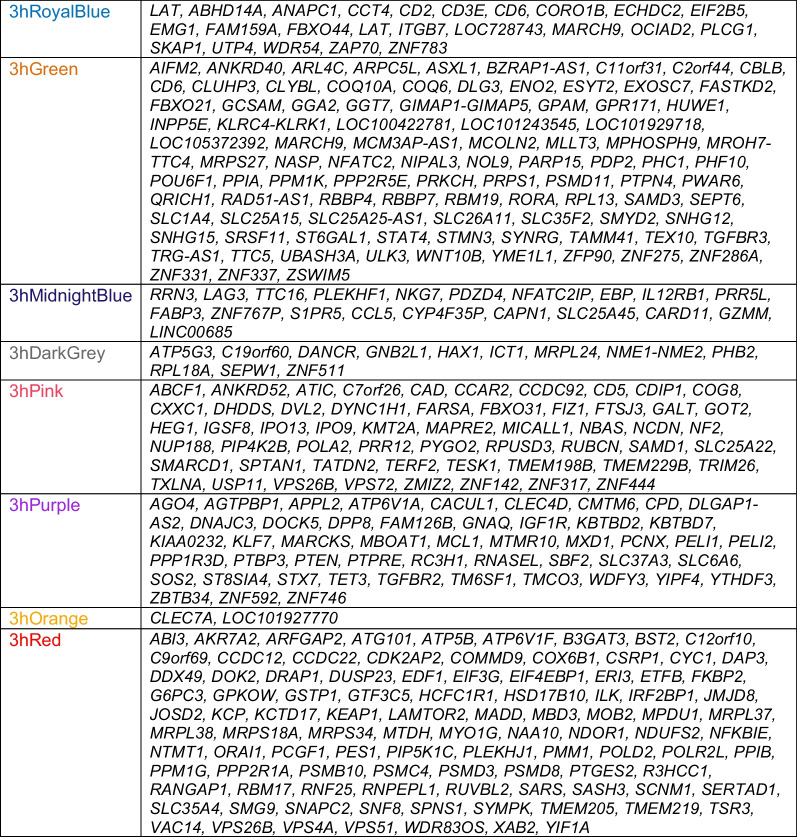
Hub genes in outcome-associated co-expression modules at ≤ 3 h from stroke onset

### Co-expressed gene modules at ≤ 3 h after IS associated with 90-day outcome

Twenty-eight co-expressed probe set modules were identified for the ≤ 3 h network (data not shown). Eight modules associated with 90-day Good and Poor outcomes (mRS) and/or 90-day NIHSS (Fig. 3b). Two outcome-significant modules, 3hPurple (Figs. 3b and 5a) and 3hOrange, positively correlated with 90d mRS. Six negatively correlated with 90-day outcome, including 3hRoyalBlue (Figs. 3b and 5b), 3hMidnightBlue and 3hDarkGrey which were significant for 90d mRS and 90d NIHSS; 3hPink for 90d mRS; and 3hGreen for 90d NIHSS (Fig. 3b). Pathway analyses for each outcome-significant module are presented in Additional file 5: Table S5A. Most outcome-significant modules and/or their hubs (Table 2) were enriched in neutrophil-, monocyte-, T cell-, and/or NK cell-specific genes (Fig. 3b). Neutrophil genes were enriched in positive-beta modules and/or hubs, while most T cell genes and/or their hubs were enriched in negative-beta modules (Fig. 3b).

The pathways for hub genes overrepresented at ≤ 3 h included T-cell pathways, calcium-induced T lymphocyte apoptosis, VEGF, NGF, neurotrophin/TRK and GDNF Signaling (Additional file 5: Table S6A). Neutrophil-specific hub genes (3hPurple module) were enriched in 39 significant pathways such as IL-2, -6 and -7, and JAK/STAT Signaling (Additional file 5: Table S6A). About half of the pathways (19/39) overrepresented in neutrophil-specific hubs were also overrepresented in the 108 pathways for T-cell specific hubs. However, the T-cell- and neutrophil-specific hubs correlated in opposite directions with 90-day outcome (Fig. 3b).

Figure 5a shows a 3-h module enriched with neutrophil-specific genes (3hPurple) and Fig. 5b for a 3-h module enriched with T-cell specific genes (3hRoyalBlue) and their top overrepresented pathways (right side of the figure). The 3hPurple module (Additional file 5: Table S5A) for good vs. poor 90d mRS outcomes showed suppression of the PPARα/RXRα pathway which regulates NF-kB signaling. For the 3hRoyalBlue module, Calcium-induced T Lymphocyte Apoptosis, IL-2 Regulation in T Lymphocytes, PKCθ Signaling in T Lymphocytes, and NFAT Regulation of Immune Responses were suppressed in Poor vs. Good outcome (Fig. 5b, Additional file 5: Table S5A).

The data for co-expressed gene modules at 5 h after IS associated with 90-day outcomes are provided in Fig. 3b, Additional file 3: Fig. S3, and Additional file 5: Tables S5B and S6B. The data for co-expressed gene modules at 24 h after IS associated with 90-day outcome are provided in Figs. 3b and Additional file 4: Fig. S4 and Additional file 5: Tables S5C and S6C.

## Discussion

Expression of genes and gene co-expression modules in peripheral blood at early times after IS correlates with 90-day outcomes. Upregulation of genes in neutrophils and down-regulation in monocytes, T cells and B cells may play a role in mediating damage and repair following stroke and ultimately affect long-term outcomes [23–25]. We found that many genes were significantly regulated at all three time-points after IS indicating that these genes and immune-related pathways were reproducible and the most likely to be replicated in future studies. The findings expand our understanding of the transcriptomic changes in immune and clotting systems associated with outcome following human IS. The identified genes may be novel targets for modulating outcome, and a subset of these genes might be developed in the future to predict outcome.

### Immune/inflammatory genes/pathways associated with poor 90-day outcome

Inflammation plays a critical role in damage and repair following stroke [26]. Specific inflammatory blood markers correlate with outcomes after stroke [27], including proinflammatory cytokines like IL-1, IL-6, TNF, as well as anti-inflammatory cytokines like TGF and IL-10 [26–28]. Increases in matrix metalloproteinases (MMPs) including MMP-9 derived mainly from neutrophils are reported to cause BBB (blood–brain barrier) damage [29] and hemorrhagic complications [30–32]. MMP-9 levels correlate with infarct volume, stroke severity, and functional outcomes [32]. In our study, MMP9 expression (≤ 3 h) was up-regulated 2.4 fold in participants with poor 90-day outcomes, which is consistent with other studies showing blood MMP-9 levels correlate with poor 90d IS outcomes [33].

S100A12 mRNA, which is highly expressed by neutrophils, was up-regulated in this study at 3 h in peripheral blood of participants with poor 90d IS outcome (FC = 2.1). Elevated S100A12 plasma levels at admission following IS have previously been associated with poor mRS outcome at 90 days [34]. In addition, S100A12 serum levels increase after traumatic brain injury (TBI) and intracerebral hemorrhage (ICH) [35, 36].

The STAT3 pathway was up-regulated at all three time-points, and several interleukin (IL)-related pathways (including IL-6 at 3 h and 5 h) were activated in participants with poor IS outcomes. STAT3 promotes inflammatory responses and IL-6 promotes phosphorylation of JAK2/STAT3. Serum IL-6 levels have previously been associated with poor long-term IS outcomes [37, 38]. p38 MAPK, also significantly activated in participants with poor 90-day IS outcome, modulates proinflammatory cytokines (IL-1β, TNF-α and IL-6) and has been proposed as a therapeutic IS target.

*SMAD4* was up-regulated in IS participants with poor 90-day outcome at all three time-points. SMAD4 has been implicated in inflammation and hypercoagulation in ischemic stroke, has been associated with BBB disruption and in our previous study was up-regulated in IS participants who later developed hemorrhagic transformation [39]. We have previously observed higher expression of *SMAD4* after IS, and particularly higher in individuals with the GG allele of rs975903 [40]. This could relate in part to post-translational regulation of SMAD proteins in response to TGF-β signaling.

Specific cytokine/chemokines such as *SPRED2*, *OSM* and *IL1A* (at all three time-points), and *CXCL6* (at 3 h post-IS) were up-regulated in participants with poor outcome, while *FLT3LG* and *CCR7* (at all three time-points) were down-regulated. SPRED proteins modulate angiogenesis, vascular repair, and autophagy [41, 42]. Thrombin aggravates astrocyte injury following IS by SPRED2 activation of autophagy pathways [42]. OSM (oncostatin M), an IL-6 cytokine family member, modulates inflammatory responses and experimental stroke outcomes [43–45]. CXCL6 (C-X-C motif Chemokine Ligand 6), a chemoattractant for neutrophils and other granulocytes, is elevated following experimental ischemia–reperfusion injury [46]. *FLT3LG* (aka FLT-3L), down-regulated in 90d poor outcomes in our study, promotes differentiation of multiple hematopoietic cell lineages. Low FLT3LF serum levels within 72 h of stroke onset have been observed in severe stroke [47]. *CCR7* (C–C motif Chemokine Receptor 7), also down-regulated in poor outcomes, activates B and T lymphocytes and regulates T cell migration to sites of inflammation and stimulates dendritic cell maturation [48, 49]. Overall, our findings in peripheral blood support the notion of complex effects of cytokines/chemokines on stroke pathophysiology and outcome. The early IS transcriptomic response in peripheral blood suggested a strong neutrophil response and activated inflammatory pathways associated with poor long-term outcomes. However, inflammation has been shown to have detrimental as well as beneficial roles following IS that are highly time-dependent [50]

### Down-regulation of lymphocyte-specific genes associated with poor 90-day outcome

Lymphocytes modulate IS in a time-dependent manner, and the peripheral transcriptome responses of lymphocytes to IS are fairly unique compared to other conditions [51]. In this study, we showed enrichment in lymphocyte-specific genes for T cells, B cells and natural killer (NK) cells in the per-gene and WGCNA analyses. Most of these genes were down-regulated in poor 90-day outcome participants. In this study several overrepresented T call pathways were found in the outcome-significant WGCNA modules as noted in the Additional file 6: Discussion.

Our data imply that down-regulation of lymphocyte-specific genes was associated with poor 90-day outcome. However, this is complicated by the fact that various studies have shown decreases of lymphocytes in blood of humans following stroke, thus partly accounting for decreases in expression. Moreover, since we investigated the changes in the transcriptome in whole blood, we can only infer cell-type specificity from known cell-specific gene expression and cannot decipher the entire transcriptomes of the specific lymphocyte cell types. Thus, additional studies of isolated peripheral blood cell types are needed to further refine the contribution of each cell type to the post-stroke response and its association with outcome.

### Coagulation, platelet, and cardiovascular pathways associated with outcome

Coagulation and platelet activation are involved in causing IS and may play a role in long-term outcomes [52]. In our study poor 90d outcomes were associated with enrichment of Thrombin pathways at 5 h and 24 h, Thrombopoietin Signaling at 5 h and the Intrinsic Prothrombin Activation Pathway at 24 h after stroke. Thrombopoietin (TPO), protective in experimental focal stroke [53], stimulates the production and differentiation of megakaryocytes and regulates platelet formation. In addition, several coagulation factors such as Factor 5 (*F5*, coagulation factor V; causative gene in Factor V Leiden thrombophilia), *F8* and *F12*, which are part of the Intrinsic Prothrombin Activation Pathway, were up-regulated at 24 h in IS participants with poor 90d outcomes. These findings support suggestions coagulation and fibrinolysis biomarkers are predictive of thrombolysis treatment outcome after IS [54]. Though our results provide evidence that early activation of peripheral coagulation pathways associate with long-term outcome, it is unclear whether this relates only to early fibrinolysis or to other effects on brain repair during recovery. Thus, further studies into their potential usefulness as treatment targets are warranted.

Cardiovascular function pathways regulated at 5-h post-stroke that correlated with 90d outcomes included Adrenomedullin signaling pathway, Renin–Angiotensin Signaling and HIF1α Signaling. Their potential role in stroke outcome is included in the Additional file 6: Discussion.

### Growth factor signaling

Several growth factor signaling pathways were associated with 90-day outcome, including Erythropoietin, Fibroblast Growth Factor (FGF), Transforming growth factor-β (TGF-β), Growth Hormone, Granulocyte–Macrophage Colony-Stimulating Factor (GM-CSF), Hepatic Growth Factor (HGF), VEGF and VEGF Family Ligand-Receptor signaling. Their possible role in modulating stroke outcomes is included in the Additional file 6: Discussion.

### Module hubs

Hubs, the most inter-connected genes in each co-expression module defined in WGCNA, are potential master regulators of gene expression. Five outcome-significant modules are highlighted in Fig. 5, Additional file 3: Fig. S3, Additional file 4: Fig. S4: two modules being enriched with T cell-specific genes and three with neutrophil-specific genes. The potential role of these hub genes in stroke outcomes is addressed in the Additional file 6: Discussion.

### Shared stroke outcome genes with stroke risk genes identified by GWAS

We found several genes differentially expressed in our analysis associated with stroke outcome that have also been found in genome-wide association studies (GWAS) to be significant stroke risk loci. For example, variants rs12579302, rs10886430, rs55983834, rs2501966, rs12426667 are stroke risk loci in differentially expressed stroke outcome genes *ATP2B*, *GRK5*, *SH3PXD2A*, *CENPQ*, *HOXC4*, respectively; and intergenic variants rs2107595 and rs1487504 are stroke risk loci in differentially expressed outcome genes *HDAC9* and *BNC2*, respectively [55]. In addition, the genome-wide significant stroke risk locus rs7974266 was upstream of differentially expressed gene *PTPN11*, rs12539561 was downstream of differentially expressed gene *PIK3CG*, and the 3-prime UTR variant rs42035 was downstream of differentially expressed outcome gene *CDK6* [55]. Another GWAS significant protein-coding variant (rs1778155) in the *PDE4DIP* gene was associated with an increased risk of stroke [56] and in our data *PDE4DIP* was up-regulated in participants with poor vs good outcome. Moreover, the intronic variant rs1842681 is a trans-eQTL for *PPP1R21*, implicated in brain plasticity and outcome after stroke [57]. In our study, *PPP1R21* was identified as a hub gene in the outcome-associated co-expression module at 5 h after stroke. A locus in *ADAM23* has been shown to correlate with stroke outcome [9], and in our study multiple *ADAM* gene family members such as *ADAM9*, *ADAM17*, *ADAM19* were associated with poor stroke outcomes. These results are of interest since they show that some of the genes associated with increased risk of stroke are also associated with long-term outcomes after stroke.

## Limitations

The findings from this study need to be validated in larger cohorts. The cell-specific genes used here [20, 21] were identified in healthy participants and may change expression patterns with disease. In addition, since changes in cell count of specific peripheral blood cell types have been reported following ischemic stroke, some of the outcome differences of expression in this study could be due to changes in different proportions of cells. The study included repeat blood draws of ischemic stroke participants at pre-treatment (≤ 3 h) and post-treatment (5 h, 24 h) time-points. Since no IS participants were untreated, the results cannot tease out the contribution of treatment to outcome in the 5 h and 24 h samples. Thus, we focused the results on the ≤ 3 h untreated time-point. Nevertheless, the results provide interesting insights. First, genes at times pre- and post-treatment correlated with 90d outcomes, with the greatest number of genes identified pre-treatment (3 h). Second, a comparison of the outcome genes to our previous study of tPA responsive genes in blood of rats with strokes from Jickling et al. [58] shows very little overlap except for 5 h good outcome vs. VRFC genes, suggesting most of 5 h and 24 h outcome genes were not related to tPA administration (overlap genes in Additional file 5: Table S8). The problem of multiple comparisons was dealt with in part by using the Benjamini–Hochberg False Discovery Rate (FDR) approach, commonly used in gene expression studies. However, we also emphasized those genes that were regulated at all three time-points, which helps show the reliability of those genes since they replicate. We also performed a linear regression to develop a pilot model for poor vs. good outcome. This model yielded 10 genes that predicted good vs. poor outcome with an area under the curve of 0.88 (Additional file 5: Table S7). Due to the very small sample size, future larger studies are needed to determine whether early changes of gene expression in blood of stroke patients can reliably and with generalizability predict 90-day outcomes. Since age was significantly different between participants with poor IS outcome and VRFC, we included age as a covariate together with vascular risk factors to account for their potential effect on outcome. However, additional variables such as technical variation, stroke subtype and infarct volume may also affect the outcome; thus future larger studies should address these and other additional clinical variables. In addition, the initial CLEAR trial was designed as a safety study. Thus, infarct volume and location were only recorded on a small number of participants. Thus, we were not able to state whether there was any correlation between infarct volume and outcome from this data set. As far as location, of the participants where location was recorded, all were carotid (half left and half right carotid) except for one vertebrobasilar. Thus, we cannot state whether there is any correlation between stroke location and outcome from this data set. No participant had the maximum mRS = 6 (deceased) at 90 days in this dataset. Therefore, the underlying biology of the most severe strokes that lead to death may not have been captured in this study. Moreover, there were only 10 participants with poor outcome. This small sample may not have captured all of the biology associated with poor outcomes and thus the findings need to be validated in larger cohorts.

## Supplementary Information

**Additional file 1: Figure S1.** Top 20 most significant pathways enriched with Differentially Expressed Genes (DEGs) at ≤ 3 h, 5 h and 24 h in participants with poor 90-day mRS IS outcome compared to good 90-day mRS IS outcome. The top 20 most significant activation or suppression relevant pathways are displayed. Blue bars indicate suppression/negative *Z*-score, and orange bars indicate activation / positive *Z*-score. Darker colors represent larger |*Z*-score|. ↑ (up arrow) represents *Z* ≥ 2, for the poor 90-day mRS IS outcome compared to good 90-day mRS IS outcome. ↓ (down arrow) represents *Z* ≤ − 2 significant suppression in the poor 90-day mRS IS outcome compared to good 90-day mRS IS outcome. The asterisk * represents a statistically significant pathway (*P* < 0.05). White cells represent activity pattern prediction of *Z* = 0 (suppression or activation status cannot be predicated). *Reg.* Regulation, *Expr.* Expression, *Lymph.* Lymphocytes

**Additional file 2: Figure S2.** Top 20 most significant pathways enriched with genes whose expression correlates with 90-day NIHSS at ≤ 3 h, 5 h and 24 h. The top 20 most significant activation or suppression relevant pathways are displayed. Blue shading indicates suppression (negative Z-score), orange indicates activation (positive Z-score), and darker colors represent larger |*Z*-score|. **↑** (up arrow) represents *Z* ≥ 2, significant activation and ↓ (down arrow) represents *Z* ≤ − 2 significant suppression in participants with worse outcome compared to participants with better 90-day outcome. The asterisk * represents a statistically significant pathway (*P* < 0.05). White cells represent activity pattern prediction of *Z* = 0 (suppression or activation status cannot be predicated). Grey represents no activity pattern available for the pathway in the IPA knowledge base. *Reg.* Regulation, *Expr.* Expression, *Lymph.* Lymphocytes, *Cyt.* Cytotoxic

**Additional file 3: Figure S3.** Network diagram (left panel) and Pathway Enrichment (right panel) for the 5hCyan module which is significant for association with 90-day mRS. The left panel network diagram shows the connectivity of hubs and genes within the module. Larger nodes with large labels are hub genes, representing potential master regulators. Genes are grey by default and colored if they are cell type specific. In the right panel, the top 20 relevant significant pathways are displayed, with the vertical line indicating a *P* = 0.05. Blue shading indicates suppression (negative *Z*-score), and orange indicates activation (positive *Z*-score), and darker color represents larger |*Z*-score|. The asterisk * represents *Z* ≥ 2 or *Z* ≤ − 2 in poor outcome compared to good outcome. Grey represents no activity pattern available for the pathway in the IPA knowledge base. *Signal.* Signaling

**Additional file 4: Figure S4.** Network diagram (**a** left panel) and Pathway Enrichment (**a** right panel) for the outcome-significant (mRS poor vs. good) for the 24hYellow module. In the left panel, the network diagram shows the connectivity of hubs and genes within the module. Larger nodes with large labels are hub genes, representing potential master regulators. Genes are grey by default and colored if they are cell type specific. In the right panel, the top 20 most relevant significant pathways are displayed. The significance threshold (*P* = 0.05) corresponds to the vertical black line. Blue shading represents suppression and orange activation with darker colors representing larger |*Z*-score|. An asterisk * represents statistically significant activity pattern prediction with *Z* ≥ 2 or *Z* ≤ − 2. In **b** the Network diagram (**b** left panel) and Pathway Enrichment (**b** right panel) for the outcome-significant (90-day NIHSS) for the 24hGreenYellow module. *IL2RB* and *CD247* are colored as T cell-specific but are also expressed in NK cells. *LAT* is colored as T cell specific, but also expressed in megakaryocytes. White cells represent activity pattern prediction of *Z* = 0 (suppression or activation status cannot be predicated). Grey represents no activity pattern available for the pathway in the IPA knowledge base. Other aspects of this figure are identical to that described for (**a**). *Cyt.* Cytotoxic, *Reg.* Regulation, *Expr.* Expression, *Lymph.* Lymphocytes, *Comm.* Communication

**Additional file 5: Table S1 A** Gene expression of genes at ≤ 3 h of ischemic stroke (IS) onset that associates with 90-day outcome. 467 Genes significantly differentially expressed between Poor 90-day mRS IS Outcome and Vascular Risk Factor Control (VRFC) (FDR < 0.05, |FC|> 2). 49 Genes significantly differentially expressed between Good 90-day mRS IS Outcome and VRFC (FDR < 0.05, |FC|> 2). 709 Genes (at ≤ 3 h) significantly differentially expressed between participants with Poor 90-day mRS IS Outcome and participants with Good 90-day mRS Outcome (*P* < 0.05, |FC|> 1.3). 538 Genes significantly associated with 90-day NIHSS Outcome (*P* < 0.005)** B** Gene expression of genes at 5 h of IS onset that associates with 90-day outcome**.** 526 Genes significantly differentially expressed between Poor 90-day mRS IS Outcome and VRFC (FDR < 0.05, |FC|> 2). 100 Genes significantly differentially expressed between Good 90-day mRS IS Outcome and VRFC (FDR < 0.05, |FC|> 2). 658 Genes significantly differentially expressed between Poor 90-day mRS IS Outcome and Good 90-day mRS Outcome (*P* < 0.05, |FC|> 1.3). 197 Genes significantly associated with 90-day NIHSS Outcome (*P* < 0.005). **C** Gene expression of genes at 24 h of IS onset that associates with 90-day outcome. 571 Genes significantly differentially expressed between Poor 90-day mRS IS Outcome and VRFC (FDR < 0.05, |FC|> 2). 35 Genes significantly differentially expressed between Good 90-day mRS IS Outcome and VRFC (FDR < 0.05, |FC|> 2). 363 Genes significantly differentially expressed between Poor 90-day mRS IS Outcome and Good 90-day mRS IS Outcome (*P* < 0.05, |FC|> 1.3). 147 Genes significantly associated with 90-day NIHSS Outcome (*P* < 0.005).** D** Genes consistently differentially expressed between ischemic stroke participants with poor 90-day mRS outcome and controls over the three time-points.** E** Genes consistently differentially expressed between ischemic stroke participants with good 90-day mRS outcome and controls over the three time-points. **F** Genes consistently differentially expressed between ischemic stroke participants with poor vs. good 90-day mRS outcome over the three time-points.** G** Genes consistently associated with 90-day NIHSS outcome over the three time-points.** Table S2 A** IPA Canonical Pathway Enrichment (*P* < 0.05) for gene expression at ≤ 3 h of ischemic stroke (IS) onset that associates with 90-day outcome**.** IPA Canonical Pathway Enrichment for 467 Genes Significant to Poor 90-day mRS Outcome as compared to VRFC. IPA Canonical Pathway Enrichment for 49 Genes Significant to Good 90-day mRS Outcome as compared to VRFC. IPA Canonical Pathway Enrichment for 709 Genes Significant to Poor 90-day mRS Outcome vs. Good 90-day mRS Outcome. IPA Canonical Pathway Enrichment for 538 genes associated with 90-day NIHSS Outcome.** B** IPA Canonical Pathway Enrichment (*P* < 0.05) for gene expression at 5 h of IS onset that associates with 90-day outcome**.** IPA Canonical Pathway Enrichment for 526 Genes Significant to Poor 90-day mRS Outcome as compared to VRFC. IPA Canonical Pathway Enrichment for 100 Genes Significant to Good 90-day mRS Outcome as compared to VRFC. IPA Canonical Pathway Enrichment for 658 Genes Significant to Poor 90-day mRS Outcome vs. Good 90-day mRS Outcome. IPA Canonical Pathway Enrichment for 197 genes associated with 90-day NIHSS Outcome.** C** IPA Canonical Pathway Enrichment (*P* < 0.05) for gene expression at 24 h of IS onset that associates with 90-day outcome. IPA Canonical Pathway Enrichment for the 571 Genes Significant to Poor 90-day mRS Outcome as compared to VRFC. IPA Canonical Pathway Enrichment for the 35 Genes Significant to Good 90-day mRS Outcome as compared to VRFC. IPA Canonical Pathway Enrichment for the 363 Genes Significant to Poor 90-day mRS Outcome vs. Good 90-day mRS Outcome. IPA Canonical Pathway Enrichment for the 147 genes associated with 90-day NIHSS Outcome. **D** IPA Canonical Pathway Enrichment (*P* < 0.05) for genes consistently differentially expressed between ischemic stroke participants with poor 90-day mRS outcome compared to controls over the three time-points. **E** IPA Canonical Pathway Enrichment (*P* < 0.05) for genes consistently differentially expressed between ischemic stroke participants with good 90-day mRS outcome compared to controls over the three time-points. **F** IPA Canonical Pathway Enrichment (*P* < 0.05) for genes consistently differentially expressed between ischemic stroke participants with poor vs. good 90-day mRS outcome over the three time-points. **G** IPA Canonical Pathway Enrichment (*P* < 0.05) for genes consistently associated with 90-day NIHSS outcome over the three time-points.** Table S3 A** DAVID Gene Ontology Enrichment (FDR *P* < 0.05) for the 467 Genes (at ≤ 3 h) Significant to Poor 90-day Outcome as compared to VRF Controls. **B** DAVID Gene Ontology Enrichment (FDR *P* < 0.05) for the 571 Genes (at 24 h) Significant to Poor 90-day Outcome as compared to VRF Controls. **Table S4** Hub genes in outcome-associated co-expression modules at 5 h and 24 h from stroke onset. Probe sets without annotated genes are excluded. **A** 5 h from Stroke Onset. **B** 24 h from Stroke Onset. **Table S5 A** IPA Canonical Pathway Enrichment (*P* < 0.05) for outcome-significant WGCNA modules using gene expression at ≤ 3 h from IS onset. 3hDarkGrey Significant to NIHSS at 90 day. 3hDarkGrey Significant to mRS at 90 day. 3hGreen Significant to NIHSS at 90 day. 3hMidnightBlue Significant to NIHSS at 90 day. 3hMidnightBlue Significant to mRS at 90 day. 3hOrange Significant to mRS at 90 day. 3hPink Significant to mRS at 90 day. 3hPurple Significant to mRS at 90 day. 3hRed Significant to mRS at 90 day. 3hRoyalBlue Significant to mRS at 90 day. 3hRoyalBlue Significant to NIHSS at 90 day. **B** IPA Canonical Pathway Enrichment (*P* < 0.05) for outcome-significant WGCNA modules using gene expression at 5 h from IS onset. 5hCyan Significant to mRS at 90 day. 5hDarkOrange Significant to mRS at 90 day. 5hDarkRed Significant to mRS at 90 day. 5hGreenYellow Significant to NIHSS at 90 day. 5hLightCyan Significant to mRS at 90 day. 5hLightGreen Significant to NIHSS at 90 day. 5hLightYellow Significant to mRS at 90 day. 5hRed Significant to NIHSS at 90 day. **C** IPA Canonical Pathway Enrichment (*P* < 0.05) for outcome-significant WGCNA modules using gene expression at 24 h from IS onset. 24hGreenYellow Significant to NIHSS at 90 day. 24hOrange Significant to NIHSS at 90 day. 24hPink Significant to NIHSS at 90 day. 24hYellow Significant to mRS at 90 day. **Table S6 A** IPA Canonical Pathway Enrichment (*P* < 0.05) for some cell-specific WGCNA hubs at ≤ 3 h. T cell receptor and other T cell-specific Hub Genes. Neutrophil-specific Hub Genes. **B** IPA Canonical Pathway Enrichment (*P* < 0.05) for some cell-specific WGCNA hubs at 5 h. T cell receptor and other T cell-specific Hub Genes. Neutrophil cell-specific Hub Genes. **C** IPA Canonical Pathway Enrichment (*P* < 0.05) for some cell-specific WGCNA hubs at 24 h. T cell receptor and other T cell-specific Hub Genes. Neutrophil cell-specific Hub Genes. Monocyte cell-specific Hub Genes. **Table S7** The 10 genes used as predictors of Poor and Good 90-day mRS Outcomes. **Table S8** Genes regulated by tPA in a rat stroke model (Jickling et al., 2010) overlapped with the 5 h and 24 h outcome genes in this study. The only significant overlap of tPA genes with outcomes genes was for 5 h good outcome vs VRFC (*P* = 0.005).

**Additional file 6:** Additional methods.

## Data Availability

The data from this study are available upon written request.
