# Supplementary figures and images for: Early peripheral blood gene expression associated with good and poor 90-day ischemic stroke outcomes
Source: J Neuroinflammation. 2023 Jan 23;20:13. doi: 10.1186/s12974-022-02680-y (PMC9869610; doi:10.1186/s12974-022-02680-y)

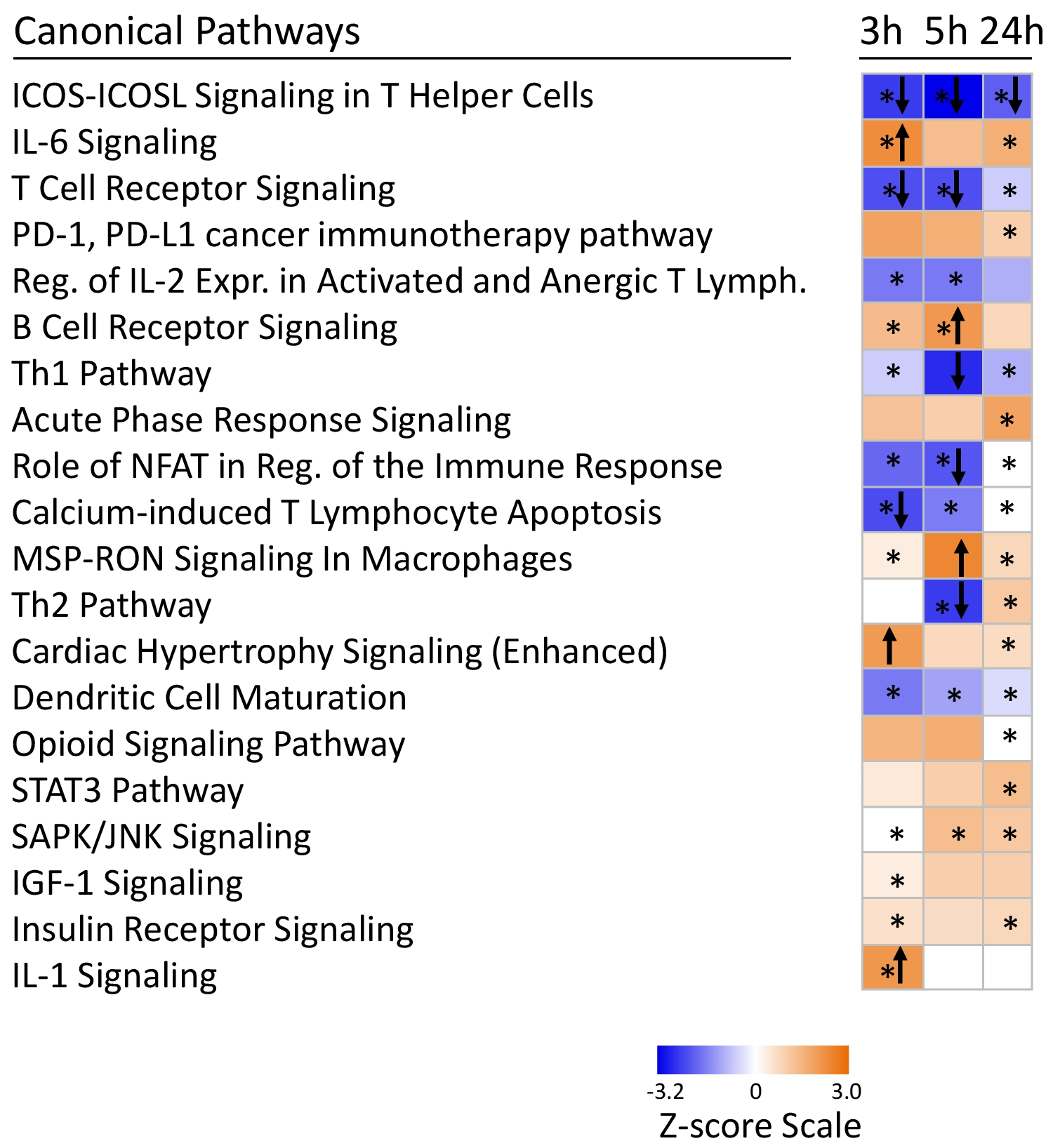

Supplement: Supplementary file 1 — Additional file 1: Figure S1. Top 20 most significant pathways enriched with Differentially Expressed Genes (DEGs) at ≤ 3 h, 5 h and 24 h in participants with poor 90-day mRS IS outcome compared to good 90-day mRS IS outcome. The top 20 most significant activation or suppression relevant pathways are displayed. Blue bars indicate suppression/negative Z-score, and orange bars indicate activation / positive Z-score. Darker colors represent larger |Z-score|. ↑ (up arrow) represents Z ≥ 2, for the poor 90-day mRS IS outcome compared to good 90-day mRS IS outcome. ↓ (down arrow) represents Z ≤ − 2 significant suppression in the poor 90-day mRS IS outcome compared to good 90-day mRS IS outcome. The asterisk * represents a statistically significant pathway (P < 0.05). White cells represent activity pattern prediction of Z = 0 (suppression or activation status cannot be predicated). Reg. Regulation, Expr. Expression, Lymph. Lymphocytes [file 12974_2022_2680_MOESM1_ESM.jpg]

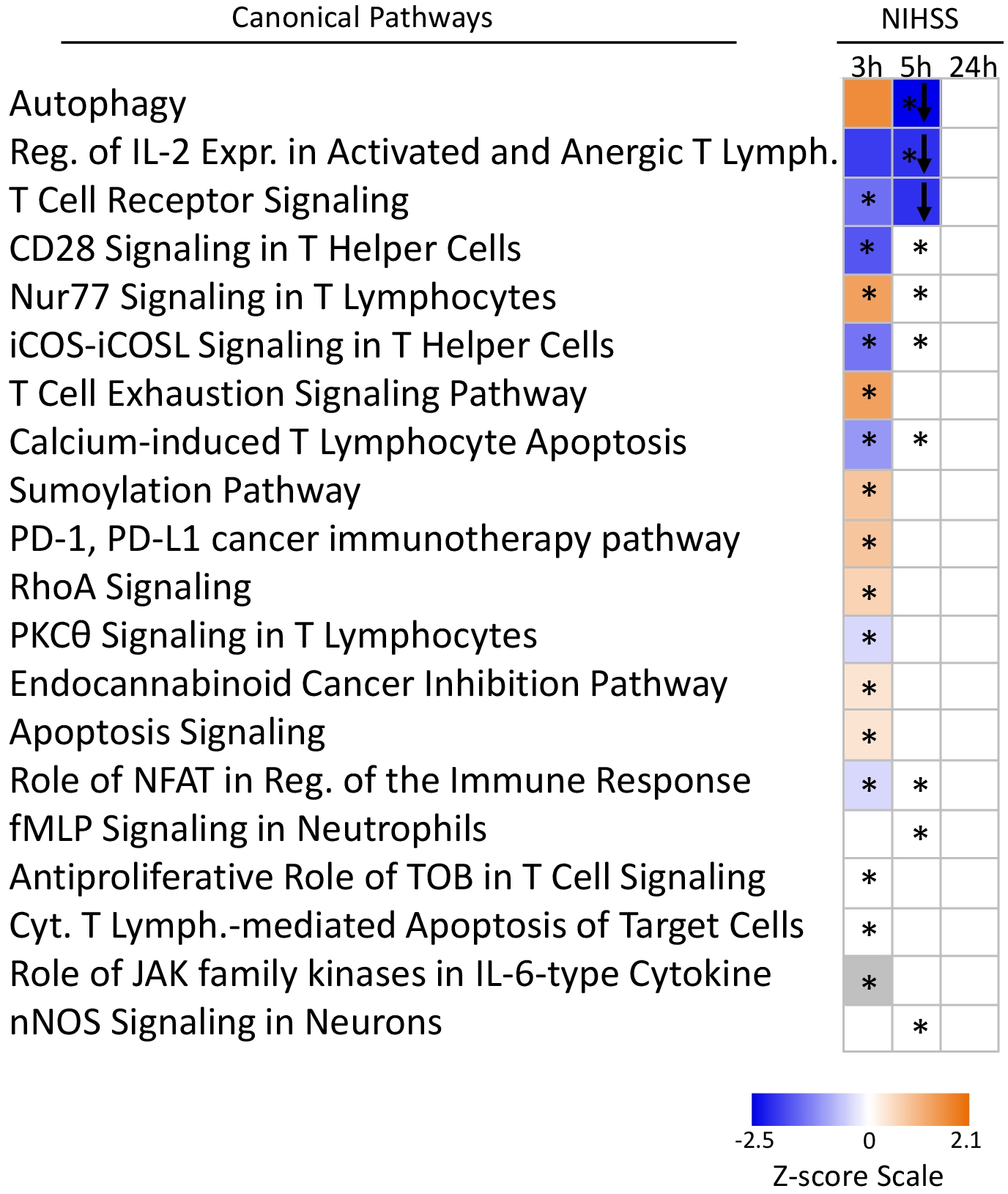

Supplement: Supplementary file 2 — Additional file 2: Figure S2. Top 20 most significant pathways enriched with genes whose expression correlates with 90-day NIHSS at ≤ 3 h, 5 h and 24 h. The top 20 most significant activation or suppression relevant pathways are displayed. Blue shading indicates suppression (negative Z-score), orange indicates activation (positive Z-score), and darker colors represent larger |Z-score|. ↑ (up arrow) represents Z ≥ 2, significant activation and ↓ (down arrow) represents Z ≤ − 2 significant suppression in participants with worse outcome compared to participants with better 90-day outcome. The asterisk * represents a statistically significant pathway (P < 0.05). White cells represent activity pattern prediction of Z = 0 (suppression or activation status cannot be predicated). Grey represents no activity pattern available for the pathway in the IPA knowledge base. Reg. Regulation, Expr. Expression, Lymph. Lymphocytes, Cyt. Cytotoxic [file 12974_2022_2680_MOESM2_ESM.jpg]

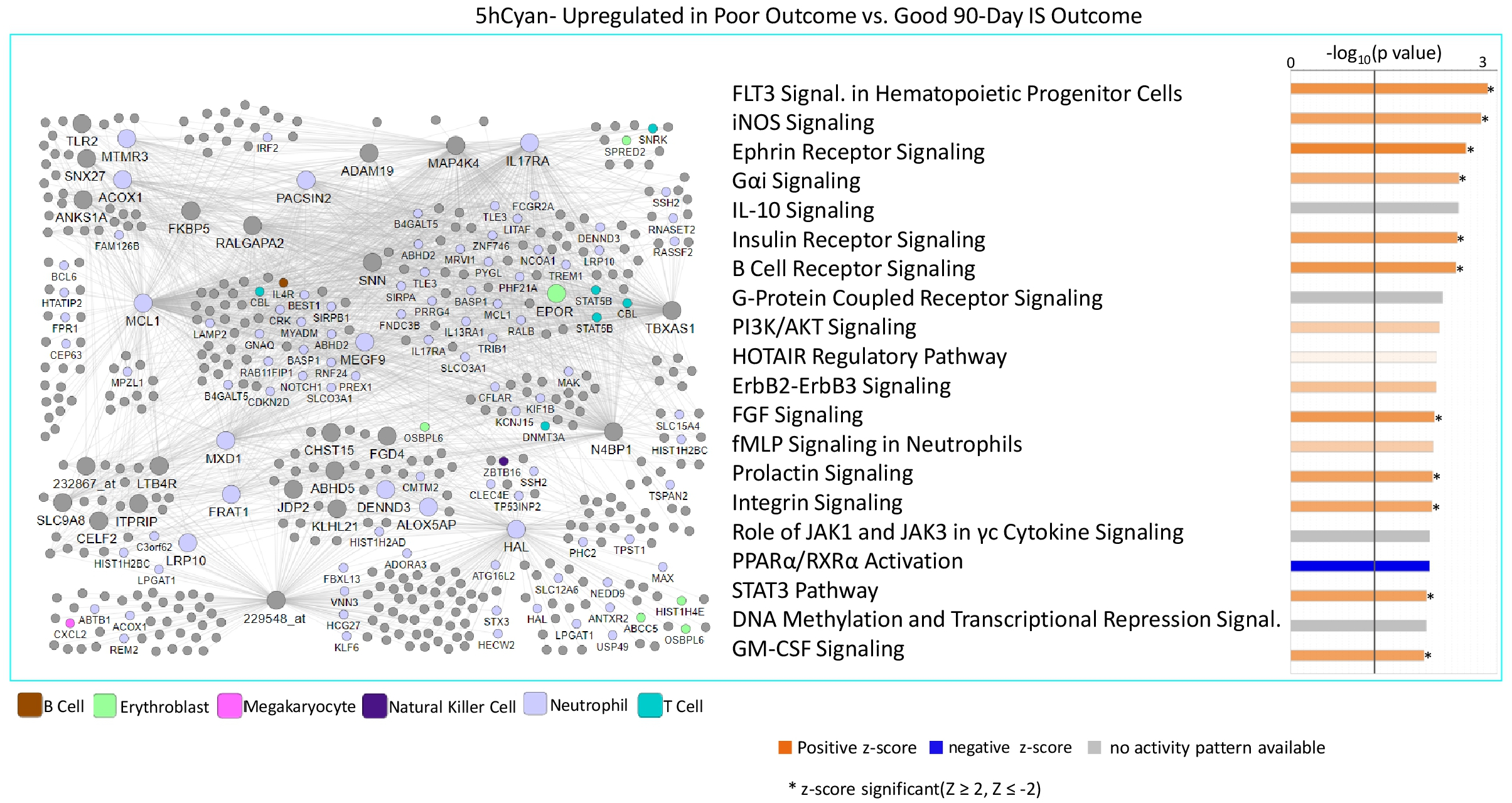

Supplement: Supplementary file 3 — Additional file 3: Figure S3. Network diagram (left panel) and Pathway Enrichment (right panel) for the 5hCyan module which is significant for association with 90-day mRS. The left panel network diagram shows the connectivity of hubs and genes within the module. Larger nodes with large labels are hub genes, representing potential master regulators. Genes are grey by default and colored if they are cell type specific. In the right panel, the top 20 relevant significant pathways are displayed, with the vertical line indicating a P = 0.05. Blue shading indicates suppression (negative Z-score), and orange indicates activation (positive Z-score), and darker color represents larger |Z-score|. The asterisk * represents Z ≥ 2 or Z ≤ − 2 in poor outcome compared to good outcome. Grey represents no activity pattern available for the pathway in the IPA knowledge base. Signal. Signaling [file 12974_2022_2680_MOESM3_ESM.jpg]

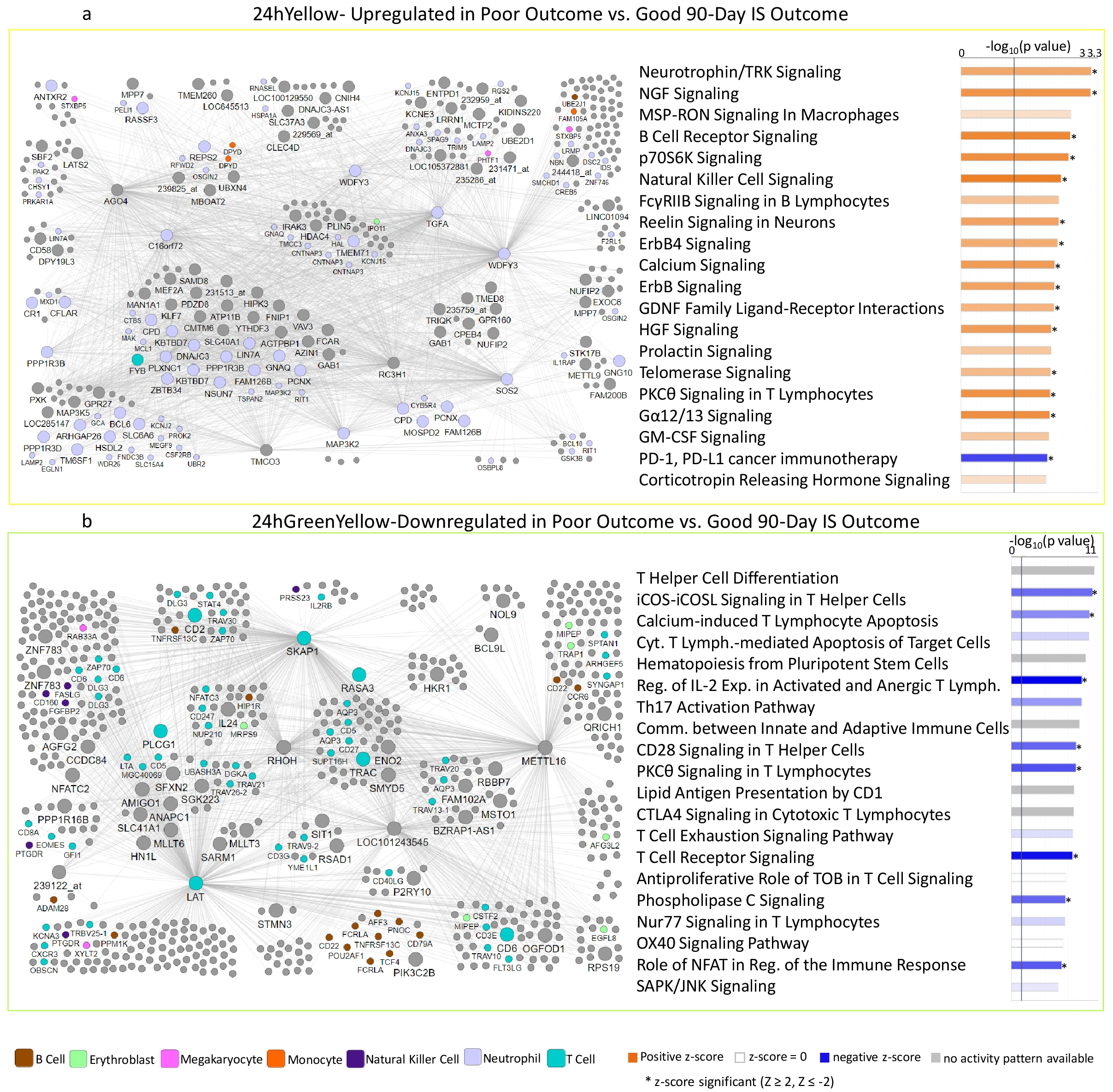

Supplement: Supplementary file 4 — Additional file 4: Figure S4. Network diagram (a left panel) and Pathway Enrichment (a right panel) for the outcome-significant (mRS poor vs. good) for the 24hYellow module. In the left panel, the network diagram shows the connectivity of hubs and genes within the module. Larger nodes with large labels are hub genes, representing potential master regulators. Genes are grey by default and colored if they are cell type specific. In the right panel, the top 20 most relevant significant pathways are displayed. The significance threshold (P = 0.05) corresponds to the vertical black line. Blue shading represents suppression and orange activation with darker colors representing larger |Z-score|. An asterisk * represents statistically significant activity pattern prediction with Z ≥ 2 or Z ≤ − 2. In b the Network diagram (b left panel) and Pathway Enrichment (b right panel) for the outcome-significant (90-day NIHSS) for the 24hGreenYellow module. IL2RB and CD247 are colored as T cell-specific but are also expressed in NK cells. LAT is colored as T cell specific, but also expressed in megakaryocytes. White cells represent activity pattern prediction of Z = 0 (suppression or activation status cannot be predicated). Grey represents no activity pattern available for the pathway in the IPA knowledge base. Other aspects of this figure are identical to that described for (a). Cyt. Cytotoxic, Reg. Regulation, Expr. Expression, Lymph. Lymphocytes, Comm. Communication [file 12974_2022_2680_MOESM4_ESM.jpg]
