# Supplementary material for: Early peripheral blood gene expression associated with good and poor 90-day ischemic stroke outcomes
Source: J Neuroinflammation. 2023 Jan 23;20:13. doi: 10.1186/s12974-022-02680-y (PMC9869610; doi:10.1186/s12974-022-02680-y)
Supplement: Supplementary file 6 — Additional file 6: Additional methods. [file 12974_2022_2680_MOESM6_ESM.docx]

**Additional file 6**

**Methods**

Gene Expression Associated with 90-day NIHSS

Separate analyses identified genes significantly correlated with 90-day NIHSS outcome using gene expression at ≤3h, 5h and 24h. *P* <0.005 was considered significant. The ANCOVA model for each time-point was Y_i_ = μ + baseline NIHSS + 24hNIHSS + 5dNIHSS + 90dNIHSS + Hypercholesterolemia + Hypertension + Diabetes + Group + Age + Sex + ε_i_. Group was either tPA or combined treatment of tPA and eptifibatide. Baseline NIHSS is NIHSS at first draw (within 3h of stroke onset); 24hNIHSS, 5dNIHSS and 90dNIHSS are the NIHSS at 24h, 5 days and 90 days, respectively.

WGCNA-1

All networks were constructed using the same 36 CLEAR trial IS patients and 28,686 probe sets. Data was imported into R and checked for missing or zero-variance probe sets using the function *goodSamplesGenes*. Pearson correlations were used to measure co-expression [1]. An approximate scale-free topology was depicted by the data. Soft thresholding powers (β) of 6, 9, and 7 were selected for the ≤3 hours network, 5 hours network, and 24 hours network, respectively, to maximize strong correlations between genes and minimize weak correlations [2]. A signed network was used to consider both positive and negative correlations [3]. The *cutreeDynamic* function (method = tree, deepsplit = 1; minimum module size = 150) was used to form modules because it can identify nested modules in complex dendrograms [4]. Hub genes, the top 5% most interconnected genes in each module, were identified by their interconnectivity (kIN—the gene’s intramodular connectivity). Hub genes may be potential master regulators within their networks [5,6].

WGCNA-2

Module association with dichotomized mRS (Poor 90-day Outcome, Good 90-day Outcome) was modeled by Y*_i_* = *μ* + dichotomized_mRS + Hypercholesterolemia + Hypertension + Diabetes + Group + Age + Sex + ε*_i_* where Y*_i_* is the module eigengene value (first principal component of expression), *μ* is the common effect for the whole experiment, dichotomized_mRS is a binary categorization of the 90-day patient outcome into Good (mRS = 0-2) and Poor (mRS =3-5), Group is the patient treatment group (tPA; tPA+eptifibatide). Module association with 90-day NIHSS as a continuous variable was modeled by Y*_i_* = *μ* + baseNIHSS + 24hNIHSS + 5dNIHSS + 90dNIHSS + Hypercholesterolemia + Hypertension + Diabetes + Group + Age + Sex + ε*_i_*. A value of *P* < 0.05 was considered significant.

Pathway Analyses

IPA’s pathway activity prediction analysis determined if the significant pathways were activated or inhibited using the expression direction (correlation coefficient or fold change) of the input genes. IPA’s Z-score algorithm calculated the predicted overall activation/inhibition states of the canonical pathways by statistically comparing our uploaded datasets with the IPA knowledge base’s expression patterns [7]. For the network analyses, since we calculated the correlation or the fold change between the 90-day NIHSS outcome and dichotomized mRS (respectively) with the module’s eigengene, and since co-expressed probe sets within each module may have different direction correlations or fold changes, we separately calculated the partial correlation and fold change (contrast Poor 90-day Outcome vs. Good 90-day Outcome) between each probe set and the particular outcome measure using the same model used to assess module significance. This correlation or fold change value was input into IPA for prediction of the pathways’ activation/suppression status. Canonical pathways with Z ≥ 2 were considered significantly activated, while ones with Z ≤ −2 were considered significantly suppressed. For modules associated with dichotomized mRS, since we input fold change for Poor vs. Good outcome, pathways predicted to be activated are predicted to be activated in participants who will have poor 90-day outcomes compared to those with good outcomes, while pathways predicted to be suppressed are predicted to be suppressed in participants who will have poor outcomes compared to participants with good outcomes. Similarly, for modules associated with 90-day NIHSS, we input correlation coefficients with NIHSS, where the higher the NIHSS value, the worse the outcome, pathways with significant activation mean that the more activated the pathways, the worse the 90-day outcome. Pathways with significant suppression mean that the more suppressed the pathway, the worse the 90-day outcome.

IPA (Ingenuity Pathway Analysis) and the DAVID Functional Annotation Bioinformatics Recourses Database were used to identify statistically significant functional categories in the data set using a modified Fisher's exact test (*P* <0.05). The threshold for the EASE score used for the gene-enrichment analysis is based on a modified Fisher's exact *P* value. Fisher's exact tests determined whether there were more genes per biological category that are differentially expressed between the groups than would be expected by chance [8,9].

Predicting 90d Outcome from Changes in Gene Expression between 3h and 24h after IS

To identify early genes to predict long-term IS outcome, we engineered a new variable for each probe set by calculating the change in gene expression between 24h and 3h post IS.

Participants were divided into a training set (n=25) and a validation set (n=11). Because of the small sample size, we undertook a two-prong approach. First, genes were excluded which upon 1-way ANOVAs were significant for Age, Sex, Hypertension, Diabetes, Hyperlipidemia, and/or Treatment Group. After excluding probe sets significant at *P*<0.05 for any of these variables, ANOVA was performed (Y = *μ* + 24hNIHSS + dichotomized _mRS + ε) on the remaining 30,565 probe sets. Probe sets with *P*<0.005 for Poor vs. Good mRS outcome were considered significant. Second, we overlapped the findings from the training set of 25 participants with the ones from the entire set of 36 and found 10 overlapping probe sets. The Δ (24h – 3h) gene expression of the 10 probe sets (features) were input into logistic regression and support vector machines (SVM) models with parameters varied as implemented in the scikit-learn package [10]. The classifier was generated from the training set and the best predictive model was deployed on the validation set. The validation set was used to evaluate the performance of the predictors by calculating the sensitivity, specificity, and Receiver Operating Characteristic (ROC) Area Under the Curve (AUC).

**Results**

Poor 90-day IS Outcome vs. Control - 24h

Seven hundred fifty-five probe sets (representing 571 genes) were differentially expressed at 24h in participants with poor outcome compared to VRFC (FDR-corrected *P* <0.05, FC> |2| (Figure 1a). Of these, 490 probe sets were up-regulated and 265 down-regulated in participants with poor outcome (Figure 1a, Table S1C). The 755 probe sets were overrepresented in 58 pathways with seven activated pathways including Regulation of The Epithelial Mesenchymal Transition by Growth Factors Pathway, STAT3 pathway, IL-1 signaling and FGF Signaling (Figure 2c, represents only the top 20 most significantly enriched pathways with significant activation or suppression Z-scores, Table S2C). The B cell receptor signaling and immunoglobulin receptor binding were overrepresented GO terms, including several genes encoding immunoglobulin heavy constant and variably chains, such as genes such as *IGHG3*, *IGHM*, *IGHG1*, *IGHV3-23*, *IGHD*, *IGHA1*, *IGHA2* (FDR < 0.05) (Table S3B). In addition, there was a significant enrichment in neutrophil-specific genes (48/571 genes (8.4%), *P*(overlap) = 1E-07); and in T cell-specific genes (12/571 genes (2.1%), *P*(overlap) = 3E-02 (Figure 3a). Notably, most of the neutrophil-specific genes were up-regulated (41/48), while most of the T cell-specific genes (9/12) were down-regulated in participants with poor 90-day functional outcome compared to controls.

Good 90-day IS Outcome vs. Control - 24h

Fifty probe sets (representing 35 genes) were differentially expressed at 24 hours after IS in participants with good 90-day outcome compared to VRFC (FDR-corrected *P* <0.05 and FC > |2|) (Figure 1a). Of these, 10 probe sets were up-regulated and 40 down-regulated in participants with good outcome (Figure 1a, Table S1C). They were overrepresented in 23 pathways (Table S2C). In addition, there was a significant enrichment in Erythroblast-specific genes (3/35 genes (8.6%), *P*(overlap) = 1.4E-02) (Figure 3a).

Association of Gene Expression with 90-day NIHSS

At ≤3h post-IS, 671 probe sets (538 genes) were associated with 90d NIHSS (*P*<0.005). 469 probe sets negatively correlated and 202 positively correlated with 90d NIHSS (Figure 1c, Table S1A). The 671 probe sets were overrepresented in 34 pathways (Table S2A) with significant enrichment with T helper cell-specific, T cell and T cell receptor signaling-specific genes (5/538 genes (0.9%), *P*(overlap) = 1E-04; 21/538 (3.9%), *P*(overlap) = 8E-07; and 13/538 (2.4%), *P*(overlap) = 2E-03, respectively) (Figure 3a). All T helper cell, T cell and T cell receptor genes except *OSBPL10* negatively correlated with 90d NIHSS. Figure S2 shows the top 20 most significant activation or suppression relevant pathways with genes whose expression correlates with 90-day NIHSS at ≤3h, 5h and 24h.

At 5h post-IS, 256 probe sets (197 genes) correlated with 90-day NIHSS (*P*<0.005). Of these 205 probe sets were negatively correlated and 51 were positively correlated (Figure 1c, Table S1B). The 256 probe sets were overrepresented in 24 pathways with two suppressed pathways including Autophagy, and Regulation of IL-2 Expression in T Lymphocytes which were significant for both overrepresentation (*P*< 0.05) and suppression (Z ≤ −2) (Figure S2, 20 most significant activation or suppression relevant pathways with genes whose expression correlates with 90-day NIHSS at 5h, Table S2B). There was a significant enrichment in T helper-specific and T cell-specific genes (3/197 genes (1.5%), *P*(overlap)=8E-04 and 11/197 (5.6%), *P*(overlap)=2E-05), respectively (Figure 3a). T helper-specific and T cell-specific genes negatively correlated with the 90-day NIHSS.

At 24h post-IS, 201 probe sets (representing 147 genes) correlated with 90-day NIHSS (*P*<0.005). Of these, 113 probe sets were negatively correlated and 88 were positively correlated (Figure 1c, Table S1C). The 201 probe sets were overrepresented in three pathways (Table S2C). In addition, there was a significant enrichment in T cell-specific genes (5/147 genes (3.4%), *P*(overlap) = 2.5E-02) (Figure 3a). All T cell genes negatively correlated with the 90-day outcome.

Ten probe sets (8 genes) were consistently associated with 90d NIHSS at three time-points (*P*<0.005) (Figure 4d). The 10 probe sets negatively correlated with 90d NIHSS (Table S1G). The 10 probe sets were overrepresented in 28 pathways such as T Cell Exhaustion Signaling Pathway, T Cell Receptor Signaling and Cytotoxic T Lymphocyte-mediated Apoptosis of Target Cells (Table S2G).

Predicting 90d Outcome from Changes in Gene Expression between 3h and 24h after IS

Since changes in gene expression over time may be more predictive of long-term outcome than gene expression at a single time-point, we calculated the difference in gene expression between 24h and 3h (Δ(24h-3h)) post-IS. We derived ten genes (Table S7) that predicted 100% of good (n=18/18) and poor outcomes (n=7/7) in the training set. They also predicted 8/8 good 90-day mRS outcomes and 2/3 poor outcomes in the validation set (n=11; ROC-AUC= 0.88). Thus, the 10 genes predicted 26/26 good outcomes and 9/10 poor outcomes overall.

**Discussion**

Down-Regulation of Lymphocyte-Specific Genes Associated with Poor 90-day Outcome

Several overrepresented T cell pathways were found in the outcome significant WGCNA modules. These included PKCθ Signaling in T Lymphocytes, CD28 Signaling in T Helper Cells, iCOS-iCOSL Signaling in T Helper Cells, Calcium-Induced T Lymphocyte Apoptosis, and the Th1 Pathway. T cell-specific hub genes in the modules included Cluster of Differentiation (*CD2*, *CD3E*, *CD5*, and *CD6*), *LAT*, *STAT3*, *STAT4*, *ZAP70*, *GZMM*, *PLEKHF1*, *PRKCH*, *TGFBR3*, *YME1L1*, *SPTAN1*, *DOK2*, *UBASH3A*, and *SKAP1*. Several of these genes have been implicated in stroke. For example, *SKAP1* (Src kinase associated phosphoprotein 1) interacts with Src Family Kinases (SFKs) and stimulates T cell antigen receptors to activate integrins [11,12]. The T cell-specific hub genes from the 3h WGCNA modules were enriched in pathways such as T Cell-Receptor Signaling and were predicted to be suppressed in participants with poor 90-day outcomes. The T cell-specific hub genes included genes associated with stroke and ones important for repair after stroke (e.g., *AQP3*, *CD40LG*, *CD28*, *CAMK4*, *DNMT3A*, *EVL*, *KCNA3*, *LCK*, *MAL*, *PDE4D*, *SPTAN1*, *ARHGEF7*, *CBL*, *PLCG1*, *PRKCB*, *STAT1*, *STAT3*, *STAT5B*) [13]. *LCK* whose expression at 3h is significantly associated with poor 90d mRS and NIHSS, is a member of SFK gene family expressed in T cells [14] and modulates outcomes in experimental ischemic stroke [15]. The cytokine *CD40LG* (CD40 ligand), also down-regulated in poor outcomes vs. good outcome, is a target of the FDA-approved drug Letolizumab (PubChem BMS-986004). Our results could indicate that up-regulation of T cells and their genes might modulate long-term outcomes.

B cells and NK cells

B cells are part of adaptive immunity and support neuronal survival, plasticity, recovery, and neurogenesis by producing proteins like neurotrophins to protect neurons [16,17]. Lack of B cells has been shown to increase stroke-induced infarct volume and mortality in mice [18]. In addition, B cell transfer reduced infarct volumes 3d and 7d after transient middle cerebral artery occlusion (tMCAO) in mice [19]. However, additional studies into the long-term role of B cells suggested B cells may contribute to cognitive decline weeks after stroke in mice [20]. Thus, their role is either detrimental or reparative depending on timing, location, and function of B cells/type of the B cells activation recruited into the injured brain [20]. In our study, we observed significant enrichment with B-cell-specific genes in one 5h (90-day NIHSS), and one 24h (90-day NIHSS) negative-beta regression modules (lower expression in worse vs. better NIHSS 90-day outcome), and in the gene lists of Poor Outcome vs. Control at 5h, and Poor vs. Good Outcome at 5h and 24h. Most of the B cell-specific genes were down-regulated in poor outcomes. On the other hand, in modules different from the ones enriched with B cell-specific genes, the B Cell Receptor Signaling was significantly activated in participants in poor vs. good 90-day mRS outcome in one 3h module, two 5h modules and one 24h module. All four modules were positive-beta regression modules for 90-day mRS (higher eigengene expression in poor vs. good outcome). In addition, B Cell Receptor Signaling was significantly suppressed in one 24h module that negatively associated with 90-day mRS, which was also enriched in B-cell specific genes. In that module, the more suppressed the B Cell Receptor Signaling Pathway, the higher the mRS, the worse the 90-day mRS outcome. Thus, our data underscore the complex involvement of B cells in the early peripheral B cell immune response to ischemic stroke. The identified genes may be potential therapeutic targets.

NK cells are a critical component of the innate immune system. They infiltrate the injured brain following stroke and are detected in the peri-infarct region [21]. They have been associated with inflammation and infections after stroke, and with exacerbation of brain infarction, BBB damage and infarct size [21]. Recruitment of NK cells by ischemic neurons has been shown to accelerate brain infarction [22]. In addition, decreased NK cell counts in peripheral blood in days one, three and seven following IS have also been reported [23]. In our data, NK-specific genes were significantly enriched in one 3h module, one 5h module (and their hubs), and one 24h module – all with negative beta regression coefficient with 90-day mRS (the lower the expression, the higher the 90-day mRS, the worse the 90-day outcome). Additional studies will need to further dissect the mechanisms and function of NK cells in the peripheral immune system and in the injured brain post stroke and how this affects outcome.

Cardiovascular Pathways Associated with Outcome IS

Cardiovascular functional pathways regulated at 5h post stroke that correlated with 90d outcomes. Plasma Adrenomedullin levels increase following IS and are an independent predictor of 3-month IS outcomes [24,25]. HIF1α Signaling, also activated at 5h in poor mRS outcome in two modules, regulates most hypoxia responsive genes [26]. HIF1α serum levels correlate with worse IS outcomes [27]. Renin-Angiotensin Signaling, modulated at 3h, 5h and 24h, was associated with 90d poor outcome participants. Renin-angiotensin (RAS) contributes to increased arterial pressure and has been associated with local cerebrovascular dysfunction [28]. In ischemic stroke, there may be an imbalance in the two opposing axes of RAS – a ‘classical axis’ and ‘alternative axis’ mediated by Angiotensin II and Angiotensin-(1–7), respectively [29]. Modulating RAS influences experimental stroke outcomes [30]. Thus, our human data suggest early changes of coagulation and cardiovascular function pathways are associated with poor long-term outcomes, and thus may be therapeutic targets.

Growth Factor Signaling

Several studies have found levels of certain growth factors correlate with good outcomes after IS [31–33]. However, growth factors can have pleiotropic and sometimes opposing effects [34,35]. For example, higher VEGF levels exacerbated hemorrhage after experimental brain arteriovenous malformations [35] and can worsen edema in experimental IS [36]. Another study showed high blood levels of FGF23 increased the risk for cardiovascular disease and stroke [34]. We previously found several growth factors signaling pathways associated with larger ICH volumes and peri-hematomal edema volumes [37]. Further studies are needed to better understand the association between early changes in growth factor signaling and long-term outcome, as our data showed a complex association with 90-day IS outcome.

Module Hubs

Hub genes in modules enriched in neutrophil-specific genes included *AGO4* (Argonaute RISC Component 4) and a paralog of neutrophil-specific *PTENP1*, *PTEN* (Phosphatase And Tensin Homolog). These have been implicated in immune-related pathways and brain injury [38–40]. The Argonaute RISC Component family responds to hypoxia [38] which can contribute to poor functional outcome [39]. PTEN protects against cerebral ischemia [40]. Another neutrophil-specific hub gene, *PELI1*, which contributes to microglial activation following subarachnoid hemorrhage [41], could have a similar role in IS. *MEGF9,* a neutrophil-specific hub gene, could also impact IS outcome [42]. *PPP1R3B*, also a neutrophil-specific hub gene, has polymorphisms associated with serum LDL-C levels that contribute to coronary artery and IS disease risk [43].

T cell-specific hub genes included *ZAP70*, *LAT*, *SKAP1*, *PLCG1*, and *CD3E*. LAT protein is phosphorylated by [ZAP70](https://en.wikipedia.org/wiki/ZAP70)/[Syk](https://en.wikipedia.org/wiki/Syk) protein [tyrosine kinases](https://en.wikipedia.org/wiki/Tyrosine_kinase) following activation of the T-cell antigen receptor (TCR) transduction pathway [44]. *ZAP70* is differentially expressed at 3h and 24h in poor outcome participants in this study, and is up-regulated in ICH patients [45]. *PLCG2* is expressed in human and mouse brain microglia. PLC enzymes like PLCG1 are key elements in signal transduction networks, with the PLCG2 P522R variant being protective against Alzheimer’s Disease [46]. *SKAP1* encodes a T cell adapter protein that promotes adhesion and degranulation, which stimulates T cell antigen receptors to activate integrins. Given the large number of hub genes identified across multiple cell types, however, there is a need to develop approaches for determining which might be the best treatment targets.

Predicting 90-day Outcome from Gene Expression Following IS

Previous studies have shown gene expression could predict improvement in NIHSS from admission to discharge [47]; age and 6h NIHSS could predict survival and functional recovery after 3 months [48]; and age and NIHSS at time of discharge could predict 90-day mRS [49]. In the current study, we focused on early gene expression to capture acute peripheral immune response to predict 90-day mRS. We engineered a new feature based on the change of expression between 24h and 3h. We identified 10 genes, whose Δ (24h – 3h) gene expression predicted 18/18 good outcome and 7/7 poor 90d outcomes in a training set, and 8/8 good outcome and 2/3 poor 90d mRS outcomes in a validation set. Among the 10 predictors were *AVPR1A* (arginine vasopressin receptor 1A), a receptor aggregation and release of coagulation factors, exacerbates brain inflammatory responses to injury and promotes BBB disruption and increases cerebral edema in brain injury. AVPR1A increases in injured brain, plasma and cerebrospinal fluid in IS, ICH, subarachnoid hemorrhage and TBI patients [50]. A SNP in another gene in the 10-gene predictor set, *MSRB3*, is associated with increased odds of stroke in Alzheimer’s Disease [51]. Another predictor was *APCDD1*, a Wnt/β-catenin Signaling inhibitor, which coordinates vascular remodeling and barrier maturation of retina blood vessels [52]. Another gene was *HIPK2*, which is a serine/threonine-protein kinase involved in the hypoxia response as a transcriptional co-suppressor of for arginine vasopressin (AVP), which mediates platelet *HIF1A* [53]. Silencing the circular RNA form of HIPK2 in neural stem cells improved functional recovery post IS [53]. The top overrepresented pathway in the 10-classifier gene set was p53 Signaling which is implicated in the regulation of cell death in stroke [54,55].

Our approach of calculating the change in gene expression between 3h and 24h post IS improved sensitivity compared to 24h alone (data not shown). Though the accuracy for predicting good 90-day outcome is excellent, the accuracy for predicting poor outcome in the validation set was modest due to the very small sample size. Nevertheless, in this study the classifier consisting of gene expression data alone had a higher accuracy than a classifier where age, sex and 24h NIHSS were used as predictors (data not shown). A model consisting of gene expression plus age, sex and 24h NIHSS did not improve the 90-day outcome prediction in comparison to using gene expression alone (data not shown). The results demonstrate the feasibility of developing gene predictors of IS outcome, though the best predictors may differ somewhat once large sample sizes are analyzed.

**References**

1. Langfelder P, Horvath S. WGCNA: an R package for weighted correlation network analysis. BMC Bioinformatics. 2008;9:559. doi.org/10.1186/1471-2105-9-559

2. Zhang B, Horvath S. A general framework for weighted gene co-expression network analysis. Stat Appl Genet Mol Biol. 2005;4(1). doi:10.2202/1544-6115.1128

3. Langfelder P. Signed vs. unsigned topological overlap matrix technical report. Published online 2013. https://horvath.genetics.ucla.edu/html/CoexpressionNetwork/Rpackages/WGCNA/TechnicalReports/signedTOM.pdf. Accessed 30 Oct 2020.

4. Langfelder P, Zhang B, Horvath S. Defining clusters from a hierarchical cluster tree: the dynamic tree cut package for R. Bioinformatics. 2008;24(5):719-20. doi:10.1093/bioinformatics/btm563

5. Langfelder P, Mischel PS, Horvath S. When is hub gene selection better than standard meta analysis?. PLoS ONE. 2013;8(4):e61505. doi:10.1371/journal.pone.0061505

6. Yang Y, Han L, Yuan Y, Li J, Hei N, Liang H. Gene co-expression network analysis reveals common system-level properties of prognostic genes across cancer types. Nat Commun. 2014;5(1):3231. doi:10.1038/ncomms4231

7. Ingenuity downstream effects analysis in IPA. Accessed April 18, 2022. http://pages.ingenuity.com/IngenuityDownstreamEffectsAnalysisinIPAWhitepaper.html

8. Huang DW, Sherman BT, Lempicki RA. Systematic and integrative analysis of large gene lists using DAVID bioinformatics resources. Nat Protoc. 2009;4:44–57. https://doi.org/10.1038/nprot.2008.211

9. Huang DW, Sherman BT, Lempicki RA. Bioinformatics enrichment tools: paths toward the comprehensive functional analysis of large gene lists. Nucleic Acids Res. 2009;37:1–13. https://doi.org/10.1093/nar/gkn923

10. Pedregosa F, Varoquaux G, Gramfort A, et al. Scikit-learn: machine learning in Python. J Mach Learn Res. 2011;12(85):2825-30. http://jmlr.org/papers/v12/pedregosa11a.html

11. Liu DZ, Waldau B, Ander BP, et al. Inhibition of Src family kinases improves cognitive function after intraventricular hemorrhage or intraventricular thrombin. J Cereb Blood Flow Metab. 2017;37(7):2359-67. doi:10.1177/0271678X16666291

12. Qiu H, Qian T, Wu T, Gao T, Xing Q, Wang L. Src family kinases inhibition ameliorates hypoxic-ischemic brain injury in immature rats. Front Cell Neurosci. 2021;15:514. https://doi.org/10.3389/fncel.2021.746130

13. Rouillard AD, Gundersen GW, Fernandez NF, et al. The harmonizome: a collection of processed datasets gathered to serve and mine knowledge about genes and proteins. Database. 2016;2016. doi:10.1093/database/baw100

14. Chtanova T, Newton R, Liu SM, et al. Identification of T cell-restricted genes, and signatures for different T cell eesponses, using a comprehensive collection of microarray datasets. J Immunol. 2005;175(12):7837-47. doi:10.4049/jimmunol.175.12.7837

15. Bae ON, Rajanikant K, Min J, et al. Lymphocyte cell kinase activation mediates neuroprotection during ischemic preconditioning. J Neurosci. 2012;32(21):7278-86. doi:10.1523/JNEUROSCI.6273-11.2012

16. Selvaraj UM, Poinsatte K, Torres V, Ortega SB, Stowe AM. Heterogeneity of B cell functions in stroke-related risk, prevention, injury, and repair. Neurotherapeutics. 2016;13(4):729-47. doi:10.1007/s13311-016-0460-4

17. Tabakman R, Lecht S, Sephanova S, Arien-Zakay H, Lazarovici P. Interactions between the cells of the immune and nervous system: neurotrophins as neuroprotection mediators in CNS injury. Prog Brain Res. 2004:385-401. doi:10.1016/S0079 6123(03)46024-X

18. Ren X, Akiyoshi K, Dziennis S, et al. Regulatory B cells limit CNS inflammation and neurologic deficits in murine experimental stroke. J Neurosci. 2011;31(23):8556-63. doi:10.1523/JNEUROSCI.1623-11.2011

19. Ortega SB, Torres VO, Latchney SE, et al. B cells migrate into remote brain areas and support neurogenesis and functional recovery after focal stroke in mice. Proc Natl Acad Sci. 2020;117(9):4983-93. doi:10.1073/pnas.1913292117

20. Doyle KP, Quach LN, Solé M, et al. B-lymphocyte-mediated delayed cognitive impairment following stroke. J Neurosci. 2015;35(5):2133-45. doi:10.1523/JNEUROSCI.4098-14.2015

21. Li Y, Zhu Z, Huang T, et al. The peripheral immune response after stroke-A double edge sword for blood‐brain barrier integrity. CNS Neurosci Ther. 2018;24(12):1115-28. doi:10.1111/cns.13081

22. Gan Y, Liu Q, Wu W, et al. Ischemic neurons recruit natural killer cells that accelerate brain infarction. Proc Natl Acad Sci U S A. 2014;111(7):2704-9. doi:10.1073/pnas.1315943111

23. Chen C, Ai QD, Chu SF, Zhang Z, Chen NH. NK cells in cerebral ischemia. Biomed Pharmacother. 2019;109:547-54. doi:10.1016/j.biopha.2018.10.103

24. Somay G, Halac GU, Uslu E, Aydin S. Plasma adrenomedullin in acute ischemic stroke. Neurosci Riyadh Saudi Arab. 2007;12(4):351-3

25. Zhang H, Tang B, Yin CG, et al. Plasma adrenomedullin levels are associated with long-term outcomes of acute ischemic stroke. Peptides. 2014;52:44-8. doi:10.1016/j.peptides.2013.11.025

26. Mitroshina EV, Savyuk MO, Ponimaskin E, Vedunova MV. Hypoxia-inducible factor (HIF) in ischemic stroke and neurodegenerative disease. Front Cell Dev Biol. 2021;9. https://doi.org/10.3389/fcell.2021.703084

27. Amalia L, Sadeli HA, Parwati I, Rizal A, Panigoro R. Hypoxia-inducible factor-1α in acute ischemic stroke: neuroprotection for better clinical outcome. Heliyon. 2020;6(6):e04286. doi:10.1016/j.heliyon.2020.e04286

28. De Silva TM, Modrick ML, Grobe JL, Faraci FM. Activation of the central renin-angiotensin system causes local cerebrovascular dysfunction. Stroke. 2021;52(7):2404-13. doi:10.1161/STROKEAHA.121.034984

29. Arroja MMC, Reid E, McCabe C. Therapeutic potential of the renin angiotensin system in ischaemic stroke. Exp Transl Stroke Med. 2016;8:8. doi:10.1186/s13231-016-0022-1

30. Barzegar M, Stokes KY, Chernyshev O, Kelley RE, Alexander JS. The role of the ACE2/MasR axis in ischemic stroke: new insights for therapy. Biomedicines. 2021;9(11):1667. doi:10.3390/biomedicines9111667

31. Larpthaveesarp A, Ferriero DM, Gonzalez FF. Growth factors for the treatment of ischemic brain injury (growth factor treatment). Brain Sci. 2015;5(2):165-77. doi:10.3390/brainsci5020165

32. Sobrino T, Rodríguez-Yáñez M, Campos F, et al. Association of high serum levels of growth factors with good outcome in ischemic stroke: a multicenter study. Transl Stroke Res. 2020;11(4):653-63. doi:10.1007/s12975-019-00747-2

33. Dordoe C, Chen K, Huang W, et al. Roles of fibroblast growth factors and their therapeutic potential in treatment of ischemic stroke. Front Pharmacol. 2021;12:853. doi:10.3389/fphar.2021.671131

34. Wright CB, Dong C, Stark M, et al. Plasma FGF23 and the risk of stroke. Neurology. 2014;82(19):1700-6. doi:10.1212/WNL.0000000000000410

35. Cheng P, Ma L, Shaligram S, et al. Effect of elevation of vascular endothelial growth factor level on exacerbation of hemorrhage in mouse brain arteriovenous malformation. J Neurosurg. 2019;132(5):1566-73. doi:10.3171/2019.1.JNS183112

36. Zhang ZG, Zhang L, Jiang Q, et al. VEGF enhances angiogenesis and promotes blood-brain barrier leakage in the ischemic brain. J Clin Invest. 2000;106(7):829-38. doi:10.1172/JCI9369

37. Durocher M, Knepp B, Yee A, et al. Molecular correlates of hemorrhage and edema volumes following human intracerebral hemorrhage implicate inflammation, autophagy, mRNA splicing, and T cell receptor signaling. Transl Stroke Res. 2021;12(5):754-77. doi:10.1007/s12975-020-00869-y

38. Wu C, So J, Davis-Dusenbery BN, et al. Hypoxia potentiates microRNA-mediated gene silencing through posttranslational modification of Argonaute2. Mol Cell Biol. 2011;31(23):4760-74. doi:10.1128/MCB.05776-11

39. Ferdinand P, Roffe C. Hypoxia after stroke: a review of experimental and clinical evidence. Exp Transl Stroke Med. 2016;8(1):9. doi:10.1186/s13231-016-0023-0

40. Li W, Huang R, Chen Z, Yan LJ, Simpkins JW, Yang SH. PTEN degradation after ischemic stroke: a double-edged sword. Neuroscience. 2014;274:153-61. doi:10.1016/j.neuroscience.2014.05.027

41. Huang XP, Peng JH, Pang JW, et al. Peli1 contributions in microglial activation, neuroinflammatory responses and neurological deficits following experimental subarachnoid hemorrhage. Front Mol Neurosci. 2017;10:398. https://doi.org/10.3389/fnmol.2017.00398

42. Brandt-Bohne U, Keene DR, White FA, Koch M. MEGF9: a novel transmembrane protein with a strong and developmentally regulated expression in the nervous system. Biochem J. 2007;401:447-57. doi:10.1042/BJ20060691

43. Li WJ, Yin RX, Huang JH, Bin Y, Chen WX, Cao XL. Association between the PPP1R3B polymorphisms and serum lipid traits, the risk of coronary artery disease and ischemic stroke in a southern Chinese Han population. Nutr Metab. 2018;15(1):27. doi:10.1186/s12986-018-0266-y

44. Martelli MP, Lin H, Zhang W, Samelson LE, Bierer BE. Signaling via LAT (linker for T-cell activation) and Syk/ZAP70 is required for ERK activation and NFAT transcriptional activation following CD2 stimulation. Blood. 2000;96(6):2181-90. https://doi.org/10.1182/blood.V96.6.2181

45. Durocher M, Ander BP, Jickling G, et al. Inflammatory, regulatory, and autophagy co-expression modules and hub genes underlie the peripheral immune response to human intracerebral hemorrhage. J Neuroinflammation. 2019;16(1):56. doi:10.1186/s12974-019-1433-4

46. Magno L, Lessard CB, Martins M, et al. Alzheimer’s disease phospholipase C-gamma-2 (PLCG2) protective variant is a functional hypermorph. Alzheimers Res Ther. 2019;11(1):16. doi:10.1186/s13195-019-0469-0

47. Meller R, Pearson AN, Hardy JJ, et al. Blood transcriptome changes after stroke in an African American population. Ann Clin Transl Neurol. 2016;3(2):70-81. doi:10.1002/acn3.272

48. König IR, Ziegler A, Bluhmki E, et al. Predicting long-term outcome after acute ischemic stroke: a simple index works in patients from controlled clinical trials. Stroke. 2008;39(6):1821-26. doi:10.1161/STROKEAHA.107.505867

49. Zhang MY, Mlynash M, Sainani KL, Albers GW, Lansberg MG. Ordinal prediction model of 90-Day modified rankin scale in ischemic stroke. Front Neurol. 2021;12:727171. doi:10.3389/fneur.2021.727171

50. Szmydynger-Chodobska J, Fox LM, Lynch KM, Zink BJ, Chodobski A. Vasopressin amplifies the production of proinflammatory mediators in traumatic brain injury. J Neurotrauma. 2010;27(8):1449-61. doi:10.1089/neu.2010.1331

51. Conner SC, Benayoun L, Himali JJ, et al. Methionine sulfoxide reductase-B3 risk allele implicated in Alzheimer’s Disease associates with increased odds for brain infarcts. J Alzheimers Dis. 2019;68(1):357-65. doi:10.3233/JAD-180977

52. Mazzoni J, Smith JR, Shahriar S, Cutforth T, Ceja B, Agalliu D. The Wnt inhibitor Apcdd1 coordinates vascular remodeling and barrier maturation of retinal blood vessels. Neuron. 2017;96(5):1055-1069.e6. doi:10.1016/j.neuron.2017.10.025

53. Wang G, Han B, Shen L, et al. Silencing of circular RNA HIPK2 in neural stem cells enhances functional recovery following ischaemic stroke. EBioMedicine. 2020;52. doi:10.1016/j.ebiom.2020.102660

54. Zhang T, Wang H, Li Q, Fu J, Huang J, Zhao Y. MALAT1 activates the P53 signaling pathway by regulating MDM2 to promote ischemic stroke. Cell Physiol Biochem*.* 2018;50(6):2216-28. doi:10.1159/000495083

55. Venna VR, Verma R, O’Keefe LM, et al. Inhibition of mitochondrial p53 abolishes the detrimental effects of social isolation on ischemic brain injury. Stroke. 2014;45(10):3101-4. doi:10.1161/STROKEAHA.114.006553
